# Herpes simplex encephalitis is linked with selective mitochondrial damage; a post-mortem and in vitro study

**DOI:** 10.1007/s00401-016-1597-2

**Published:** 2016-07-25

**Authors:** Małgorzata Wnęk, Lorenzo Ressel, Emanuele Ricci, Carmen Rodriguez-Martinez, Julio Cesar Villalvazo Guerrero, Zarini Ismail, Colin Smith, Anja Kipar, Beate Sodeik, Patrick F. Chinnery, Tom Solomon, Michael J. Griffiths

**Affiliations:** 1 Brain Infections Group, Department of Clinical Infection, Microbiology and Immunology, Institute of Infection and Global Health, University of Liverpool, Liverpool, L69 7BE UK; 2 Department of Infection Biology, Institute of Infection and Global Health, University of Liverpool, Liverpool Science Park IC2, Liverpool, L3 5RF UK; 3 Veterinary Pathology, School of Veterinary Science, University of Liverpool, Leahurst Campus, Neston, CH64 7TE UK; 4 Institute of Virology, Hannover Medical School, 30625 Hannover, Germany; 5 German Centre for Infection Research (DZIF), Hannover, Germany; 6 Academic Neuropathology, Centre for Clinical Brain Sciences, University of Edinburgh, Edinburgh, EH16 4SB UK; 7 Institute of Veterinary Pathology, Vetsuisse Faculty, University of Zurich, 8057 Zurich, Switzerland; 8 Institute of Genetic Medicine, Newcastle University, Newcastle upon Tyne, NE1 3BZ UK; 9 Department of Neurology, The Walton Centre NHS Foundation Trust, Fazakerley, Liverpool, L9 7LJ UK; 10 National Institute for Health Research, Health Protection Research Unit in Emerging and Zoonotic Infections, University of Liverpool, Liverpool, L69 7BE UK; 11 Department of Neurology, Alder-Hey Children’s NHS Foundation Trust, West Derby, Liverpool, L12 2AP UK

**Keywords:** Human, Herpes simplex virus encephalitis, Mitochondria, Minocycline, Gene-expression

## Abstract

Herpes simplex virus type-1 (HSV-1) encephalitis (HSE) is the most commonly diagnosed cause of viral encephalitis in western countries. Despite antiviral treatment, HSE remains a devastating disease with high morbidity and mortality. Improved understanding of pathogenesis may lead to more effective therapies. Mitochondrial damage has been reported during HSV infection in vitro. However, whether it occurs in the human brain and whether this contributes to the pathogenesis has not been fully explored. Minocycline, an antibiotic, has been reported to protect mitochondria and limit brain damage. Minocycline has not been studied in HSV infection. In the first genome-wide transcriptomic study of post-mortem human HSE brain tissue, we demonstrated a highly preferential reduction in mitochondrial genome (MtDNA) encoded transcripts in HSE cases (*n* = 3) compared to controls (*n* = 5). Brain tissue exhibited a significant inverse correlation for immunostaining between cytochrome c oxidase subunit 1 (CO1), a MtDNA encoded enzyme subunit, and HSV-1; with lower abundance for mitochondrial protein in regions where HSV-1 was abundant. Preferential loss of mitochondrial function, among MtDNA encoded components, was confirmed using an in vitro primary human astrocyte HSV-1 infection model. Dysfunction of cytochrome c oxidase (CO), a mitochondrial enzyme composed predominantly of MtDNA encoded subunits, preceded that of succinate dehydrogenase (composed entirely of nuclear encoded subunits). Minocycline treated astrocytes exhibited higher CO1 transcript abundance, sustained CO activity and cell viability compared to non-treated astrocytes. Based on observations from HSE patient tissue, this study highlights mitochondrial damage as a critical and early event during HSV-1 infection. We demonstrate minocycline preserves mitochondrial function and cell viability during HSV-1 infection. Minocycline, and mitochondrial protection, offers a novel adjunctive therapeutic approach for limiting brain cell damage and potentially improving outcome among HSE patients.

**Electronic supplementary material:**

The online version of this article (doi:10.1007/s00401-016-1597-2) contains supplementary material, which is available to authorized users.

## Introduction

Herpes simplex virus (HSV) is one of the most common causes of viral encephalitis in western countries. HSV-1 type 1 (HSV-1) is the predominant cause of herpes simplex encephalitis (HSE). Despite treatment with aciclovir, HSE is associated with 20 % mortality, and two-thirds of survivors never regain normal central nervous system (CNS) function. Improved understanding of the pathogenesis of HSE and more effective treatments are required [47, 52].

Multiple studies have described the brain pathology in human HSE cases and animal models of HSV-1 infection. Less is known about the molecular mechanisms underlying this damage. Studies examining the pathogenesis of HSE have tended to use animal in vivo and in vitro models or transformed human cell lines derived from cells outside the brain [13, 20, 45]. Phylogenetically and/or functionally distinct from the human brain, such models may not accurately reflect human HSE.

Damage to mitochondria could significantly contribute to the pathogenesis of HSE. Mitochondria are essential for energy production and cell survival [15]. They contain their own genome, the mitochondrial DNA (MtDNA). MtDNA encodes for proteins, such as cytochrome c oxidase subunit 1 (CO1), which compose key mitochondrial enzymes, including cytochrome c oxidase (CO), involved in energy production [15, 37]. In vitro studies, largely using non-neuronal or transformed cell lines, have shown that HSV-1 interacts with mitochondria, including migration of viral proteins into the mitochondria, and can cause depletion of mitochondrial DNA, RNA and proteins, and impair mitochondrial activity [13, 33, 40, 45]. However, whether mitochondrial damage occurs in the human brain, and whether this drives brain cell death has not been fully explored.

Minocycline, an antibiotic, has been reported to reduce damage in CNS models of ischaemic stroke, neuro-degeneration, traumatic brain injury and viral infection [38, 39, 42, 53–55]. Two small clinical trials have previously indicated minocycline improves patient outcome in acute stroke. However, more recent trials did not show any efficacy [29, 32, 41]. Trials are ongoing in Japanese encephalitis and neurodegenerative diseases [19, 28]. Minocycline has not always shown benefit, and there are reports of minocycline exacerbating brain damage in animal models, including for hypoxic brain injury, Parkinson’s disease and Huntington’s disease [53]. Nevertheless, the findings suggest minocycline may be worth investigating as an adjunct in HSE. Minocycline is reported to limit damage through a variety of mechanisms, including mitochondrial protection, anti-inflammatory, anti-apoptotic and anti-viral properties [27, 53].

To identify key host responses perturbed during HSE, we examined genome-wide transcript abundance in post-mortem brain tissue from human HSE cases and controls. Observing a marked loss of mitochondrial transcripts among HSE cases, we examined whether the reduction of the mitochondrial protein (CO1) was topographically linked to the presence of HSV-1 proteins in the HSE brain tissue. To determine whether HSV triggered mitochondrial damage we examined HSV-1 DNA abundance, mitochondrial enzyme activity and cell viability during the infection time-course using primary human astrocytes. Primary human astrocytes were used because they are infected during human HSE, their dysfunction contributes to neuronal death, and they can be more reliably grown in vitro compared to primary human neurons [21, 46]. To test whether MtDNA encoded enzyme activity is impaired before nuclear DNA encoded enzyme activity, we compared CO and succinate dehydrogenase function (the latter encoded entirely nuclear DNA encoded). Next, we used transmission electron microscopy to identify whether mitochondrial disruption preceded nuclear disruption. Directed by the observed mitochondrial damage, we examined whether minocycline could protect mitochondria during HSV-1 infection.

## Materials and methods

Post-mortem brain tissue was obtained from Thomas Willis Brain Bank, Oxford University. The Thomas Willis Brain Bank review board gave ethical approval for this study.

For histopathological analysis, pre-mounted, 10 µm thick serial sections of formalin-fixed paraffin-embedded tissue from six brain regions (frontal, amygdala, thalamus, hippocampus, cingulate gyrus and insular cortex) of adult HSE cases (*n* = 3) and control subjects (*n* = 5) with a cause of death not related to encephalitis (i.e. pneumonia or myocardial infarction) were stained with haematoxylin and eosin (H&E). Patient clinical information is summarised in Supplementary table S1 (Online Resource 1).

Immunoperoxidase (IP) staining for HSV-1 and CO1 was performed on consecutive sections derived from the same anatomic region and capturing the same field of view. IP staining against a single target antigen provided the most consistent results across the tissue regions. After enzymatic antigen retrieval with citrate buffer (pH 6.0), slides were incubated with primary rabbit anti-HSV-1 polyclonal antibody [1:800 dilution in Tris buffered saline with 0.05 % Tween 20 (TBST); Abcam] and primary mouse anti-CO1 monoclonal antibody (clone 1D6E1A8; 1:200 dilution in TBST; Abcam). Dako EnVision plus^®^ detection system, containing secondary goat anti-rabbit and anti-mouse antibodies and 3′3′9-diaminobenzidine tetrahydrochloride (DAB) as chromogen, was employed for visualisation (dilutions were made following the manufacturer’s instructions).

Dual immunofluorescent (IF) staining against HSV-1 and translocase of the outer mitochondrial membrane 70a (TOMM70) with DAPI as nuclear counter-stain, plus double IP staining for CO1 and HSV-1 were also performed on the tissue. Methods are documented in Online resource 2.

For the microarray studies, total RNA was extracted from 5 mm^2^ blocks of FFPE tissue. Tissue was de-paraffinised with d-limolene, rehydrated with ethanol, incubated with proteinase K for 18 h at 55 °C, and homogenised. Total RNA was extracted using Trizol (Life-Technologies) and treated with TURBO-DNase (Applied Biosystems) following the manufacturers’ instructions. Total RNA (500 ng per subject) was amplified using genome-wide Transplex Whole Transcriptome Amplification Kit (Sigma-Aldrich). Amplified RNA was labelled with Cyanine-3 via the non-enzymatic Universal Linkage System (Kreatech). Labelled RNA (200 ng) was hybridised to SurePrint G3 GE 8 × 60 K (Design ID 030495) human-specific microarrays, and scanned using G2505C scanner following the manufacturers’ instructions (Agilent Technologies). The array was custom designed to include 398 transcripts (includes replicates) encoding for 33 MtDNA encoded genes. Raw fluorescent intensity was measured and initial quality control assessment undertaken using Agilent Feature Extraction software (FE 10.5.1.1). Transcripts were analysed as previously described [24]. Briefly, background subtracted fluorescent intensity for each gene-probe was median centred, with transcripts (*n* = 2882) exhibiting >0.8 standard deviations in variation of abundance across the samples selected using Cluster 3.0, and visualised as a heat-map using Treeview 1.60 (http://www.eisenlab.org). Significant changes in transcript abundances [<5 % false detection rate (FDR)] were identified using SAM 4.0 (http://www-stat.stanford.edu/~tibs/SAM). Gene ontology over-representation analysis was undertaken among significantly and differentially expressed transcripts using the over-representation analysis program within InnateDB (http://www.innatedb.com) [6]. Significant over-representation was calculated via a hypergeometric algorithm using Benjamini Hochberg correction [25]. Gene-expression data is available at GEO (http://www.tobeconfirmed).

In vitro cell culture: Primary human astrocytes (ScienCell Research Laboratories) were cultured in Dulbecco’s modified Eagle’s medium-F12 (Sigma-Aldrich) with 10 % fetal bovine serum, 1 % l-glutamine and 1 % penicillin/streptomycin solution (Sigma-Aldrich). In all experiments astrocytes were at confluence and between passages 4 and 5. HSV-1 strain SC16 (propagated in Vero cells) was inoculated at a multiplicity of infection (MOI) of 0.01. Viral replication and mitochondrial activity were monitored 6–72 h post infection (pi). When testing pharmacological agents, minocycline (Sigma-Aldrich) was added at a final concentration of 60 µM, 1 h prior to HSV-1 inoculation and 24, 48 and 72 h pi. Examining minocycline in combination with aciclovir (Sigma-Aldrich), both drugs were added at a final concentration of 20 µM at the same time-points. In the latter experiments, a lower concentration of minocycline was used in combination with aciclovir, this concentration was more in-line with final concentrations achievable in the cerebrospinal fluid in humans [1, 22]. A minimum of three experimental replicates were performed.

Quantitative PCR (qPCR) in cell cultures: total RNA was extracted using Qiaquick RNAeasy kit (Qiagen). cDNA was synthesised from 1 µg of total RNA using RetroScript kit (Ambion). Viral DNA was extracted using Qiaquick viral kit (Qiagen). qPCR was performed with 50 ng of cDNA or 1 µl of viral DNA using TaqMan Gene-Expression Assays (Applied Biosystems) in a BioRad qPCR system following the manufacturer’s assay and thermo-cycler set-up instructions. Relative target transcript abundance for mitochondrial cytochrome oxidase 1 (CO1), tumour necrosis factor (TNF), interleukin 6 (IL-6), Caspase 3 (CASP3), B cell lymphoma 2 (BCL-2), BCL-2 associated X protein (BAX) was quantified using the *δC*_T_ method [50]. Change in HSV-1 abundance over time was expressed as the PCR fractional cycle number at which threshold fluorescence was achieved (*C*_T_). Differences in relative transcript abundance (*C*_T_, *δC*_T_ or *δδC*_T_) in the target gene was compared between exposed and non-exposed (i.e. HSV-1 infection or minocycline) across multiple time-points using a two-way repeated measures analysis of variance (ANOVA). Time-point was the row factor (repeated measure) and exposure of the column factor. When a single time-point was compared, the Mann–Whitney *U* test was employed. DAD1 (defender against cell death 1), which exhibited relatively invariable abundance between HSE cases and controls in the array data, which was used as the ‘house-keeping’ gene.

### qPCR in brain tissue

cDNA was synthesised from 500 ng of total RNA. Changes in CO1 were expressed as 1/*C*_T_. Difference in 1/*C*_T_ for CO1 was compared across three brain regions (cingulate, amygdala and frontal), two brain sections per region, among cases and controls using a two-way repeated measures ANOVA. Tissue section was the row factor (repeated measure), and case or control the column factor. Statistical analysis and plot generation were performed using GraphPad Prism 6.0 (GraphPad software). Primer sequences are provided in Online Resource 1.

Enzyme activity for two mitochondrial enzymes, CO and succinate dehydrogenase (SDH), was sequentially examined in monolayers of non-fixed astrocytes as previously described [11]. In cells with intact CO function, DAB is oxidised to a brown end-product that saturates the organelles. Cell saturation with DAB prevents accumulation of the product of SDH activity [reduced Nitro Blue Tetrazolium (NBT)]. In cells with impaired CO function, but intact SDH function, NBT is reduced to a blue end-product that accumulates inside the cell. The proportion of astrocytes exhibiting CO function was quantitatively assessed using ImageJ (http://imagej.nih.gov/ij/). Images of the astrocyte monolayer were captured at 20× magnification using a CETI Inverso T100 inverted microscope (Medline Scientific). Measurements were initially set to measure the overall picture area (analyse: set measurements: select area and integrated density; then analyse: measure). The ImageJ thresholding tool was then used to manually adjust image brightness, saturation and hue (image: adjust: threshold) to select cells positively stained for DAB. The proportion (%) of the cells stained for DAB was calculated with the overall picture area as 100 %. At least three fields of view per experimental group at each time-point from three independent experiments were examined. To mitigate analysis bias, masked images were also examined by two additional independent technicians. They ranked the images by area of monolayer positively stained for DAB.

Dual IF labelling for HSV-1 and CO1 was performed on astrocyte pellet monolayers fixed with 4 % paraformaldehyde. A primary rabbit anti-HSV-1 polyclonal antibody (1:400 dilution in TBST; Abcam) and primary mouse anti-CO1 monoclonal antibody (clone 1D6E1A8; 1:100 dilution in TBST; Abcam) were employed. Secondary anti-rabbit polyclonal antibody conjugated with DyLight549 (1:200 dilution in TBST; MenaPath) and secondary anti-mouse polyclonal antibody conjugated with DyLight488 (1:200 dilution in TBST; AbDSerotec) were used to obtain red and green fluorescence, respectively. Nuclei were counterstained with blue fluorescent DAPI nucleic acid stain (Life-Technologies). Images (four fields of view per pellet) were examined using an Eclipse 80i epi-fluorescent microscope (Nikon). The examiner was blinded to infection exposure and experimental time-point. Among DAPI positive cells, the number of cells stained for CO1 and/or HSV-1 was counted.

For transmission electron microscopy, pellets of astrocytes (recovered from the infection time-course) were fixed with 2.5 % glutaraldehyde (in 0.2 M sodium cacodylate buffer) and 1 % aq osmium tetroxide, stained with 2 % uranyl acetate and dehydrated. Cells were embedded in epoxy resin (TAAB) and polymerised at 60 °C overnight. 75 nm sections contrasted with lead citrate and uranyl acetate were examined using a Philips EM208S (FEI) at 80 kV with an ES500 W Gatan charge-coupled camera (Erlangshen). Twenty (5 per group: non-infected and 24, 48, 72 h pi) ultra-photographs (taken at 8000× magnification) were examined. The examiner was blinded to which group the images belonged. The number of normally conformed mitochondria inside the cells was quantified. Normally conformed mitochondria were defined as electron-dense elongated organelles with identifiable double membrane and multiple cristae without ultra-structural pathological features.

Dual glial fibrillary acidic protein (GFAP) and HSV-1 IF labelling, as well as Trypan Blue staining were also performed on cultured astrocytes (Methods; Online resource 3).

## Results

**Fig. 1:**
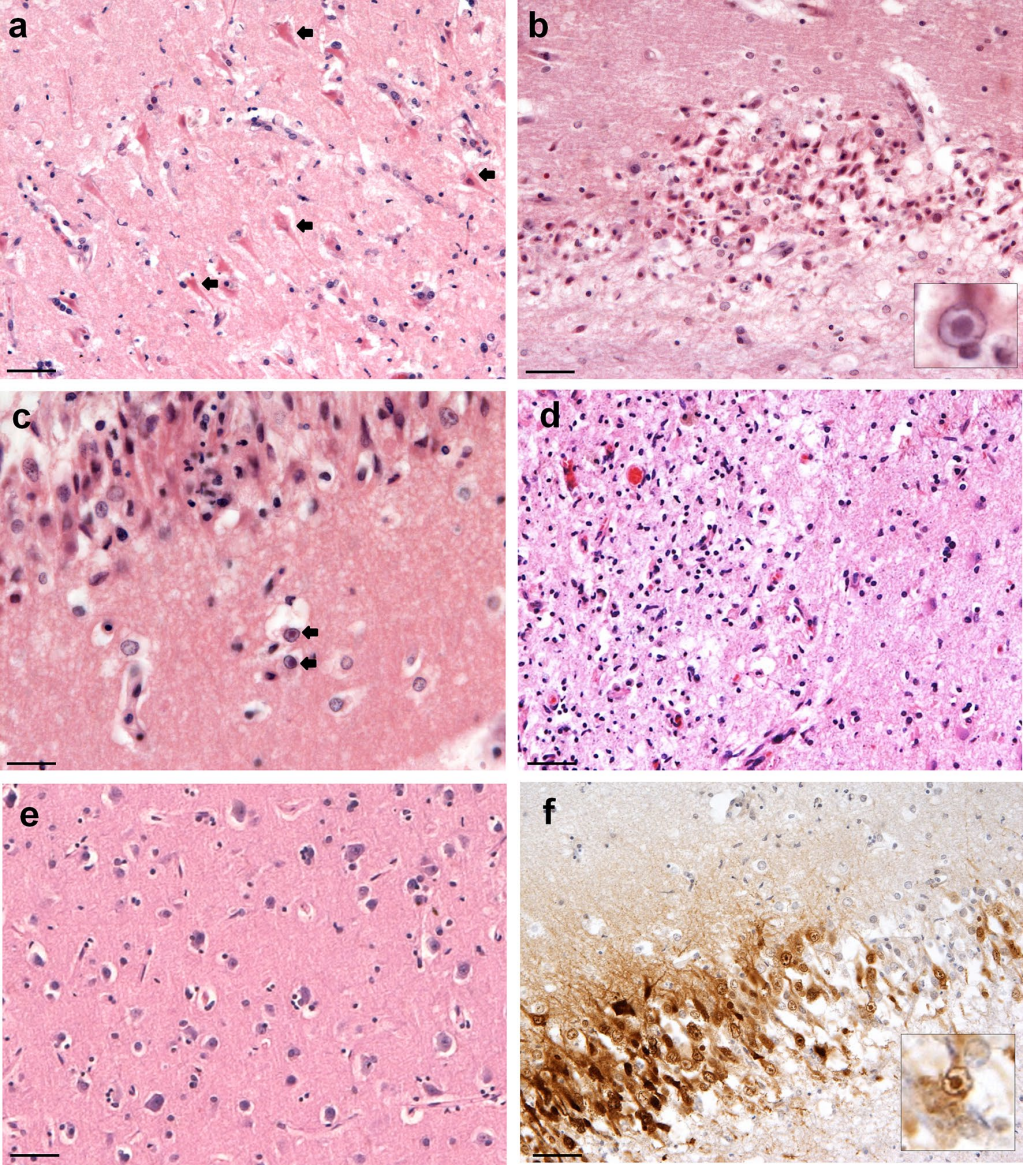
Histological features of HSE: **a** HSE patient, histological features of pyknotic and karyolytic pyramidal neurons (*arrows*). **b**, *inset* neuronal necrosis and parenchymal rarefaction in association with astrocytes containing intranuclear eosinophilic inclusion bodies. **c** Early degenerative and inflammatory changes of the grey matter associated with two astrocytes containing intranuclear inclusion bodies (*arrows*). **d** Discrete area of grey matter rarefaction with marked inflammatory cell infiltrate. **e** Control RTA patient showing unaltered neurons. **f** *Inset* hippocampus, dentate gyrus, immunoperoxidase HSV-1 antigen stain [*brown* (DAB)] is largely restricted to nuclei.* Inset image* astrocyte with enlarged dense HSV-1 staining of the nucleus. *Scale bars* 50 μm (**a**, **b**, **d**–**f**), 20 µm (**c**)

H&E stained HSE tissue sections [6 brain regions per patient (*n* = 3)] demonstrated a necrotizing and non-suppurative polioencephalitis with micro-haemorrhages and occasional parenchymal rarefaction with gitter cell infiltration and gliosis. Pyknotic and karyolytic hyperoeosinophilic neurons (Fig. 1a) were frequently observed in association with numerous astrocytes containing intranuclear eosinophilic (Cowdry type A) inclusion bodies (Fig. 1b, c). Extensive inflammatory infiltrates and haemorrhages, that effaced and replaced grey matter, were more frequently seen in frontal and temporal cortices (Fig. 1d). No significant neuropathological changes were observed in the control tissue (Fig. 1e). HSV-1 antigen staining of hippocampal HSE tissue confirmed, the astrocyte intranuclear inclusions were composed of viral particles (Fig. 1f). Further details of the pathological features observed among the HSE patients and brain regions are summarised in Table S2 (Online resource 1).

**Fig. 2:**
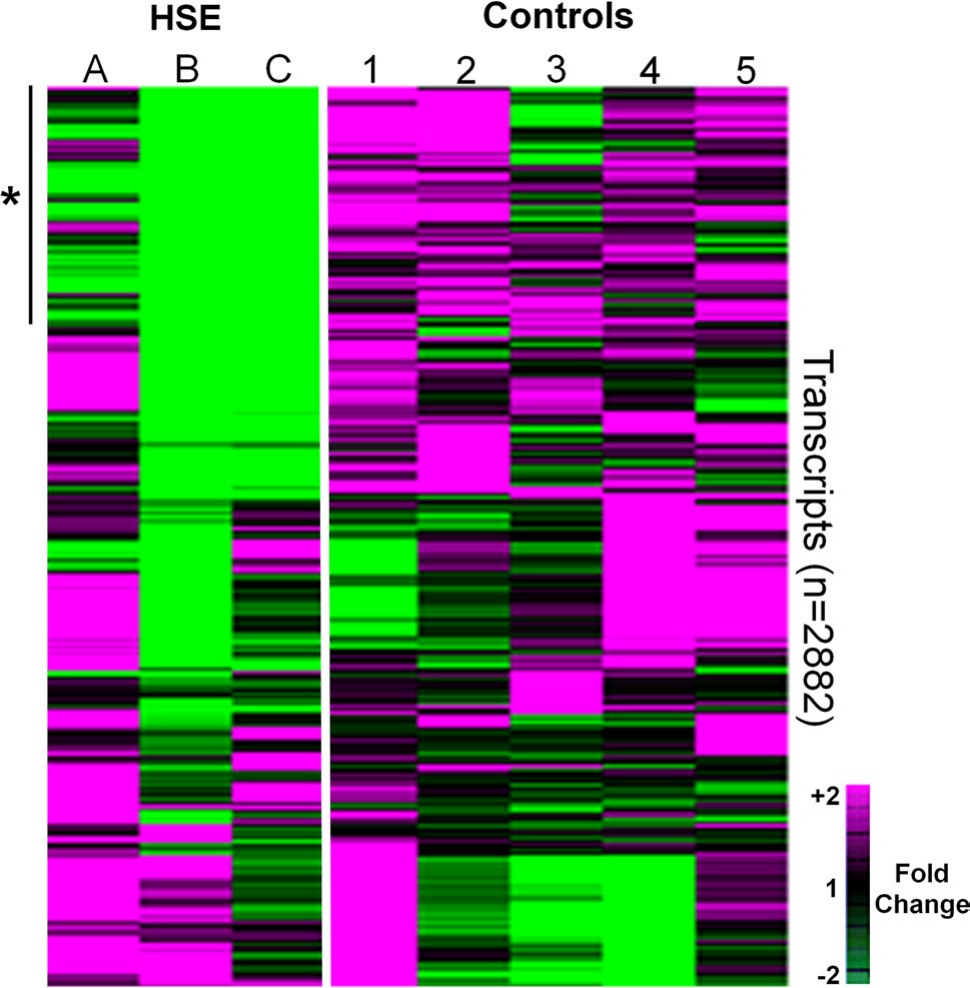
Heat-map of relative transcript abundance in the brain of HSE cases and controls. Samples are manually organised on the *x* axis; *A*–*C* represent HSE patients,* 1–5* represent control patients. Transcripts are hierarchically clustered by similarity of transcript abundance on the *y* axis (*n* = 2882). Transcripts with high abundance (relative to median value for the set) are exhibited as magenta tiles; those with low abundance—*green*; and those at median—*black*. Although there was variation in transcript abundance between the HSE cases, with cases *B* and *C* exhibiting a greater proportion of down-regulated transcripts compared to case *A*, there remained a cluster of 287 transcripts [*green tiles* in the *top left* of the heat-map (*asterisk*)] that exhibited significantly (FDR <5 %) lower abundance in HSE cases (*n* = 3) compared to controls (*n* = 5). This set of transcripts was significantly (*p* < 0.0001) over-represented with transcripts corresponding to mitochondrial DNA encoded genes. Gene-expression was visualised as a heat-map using Treeview 1.60 (http://www.eisenlab.org)

**Fig. 3:**
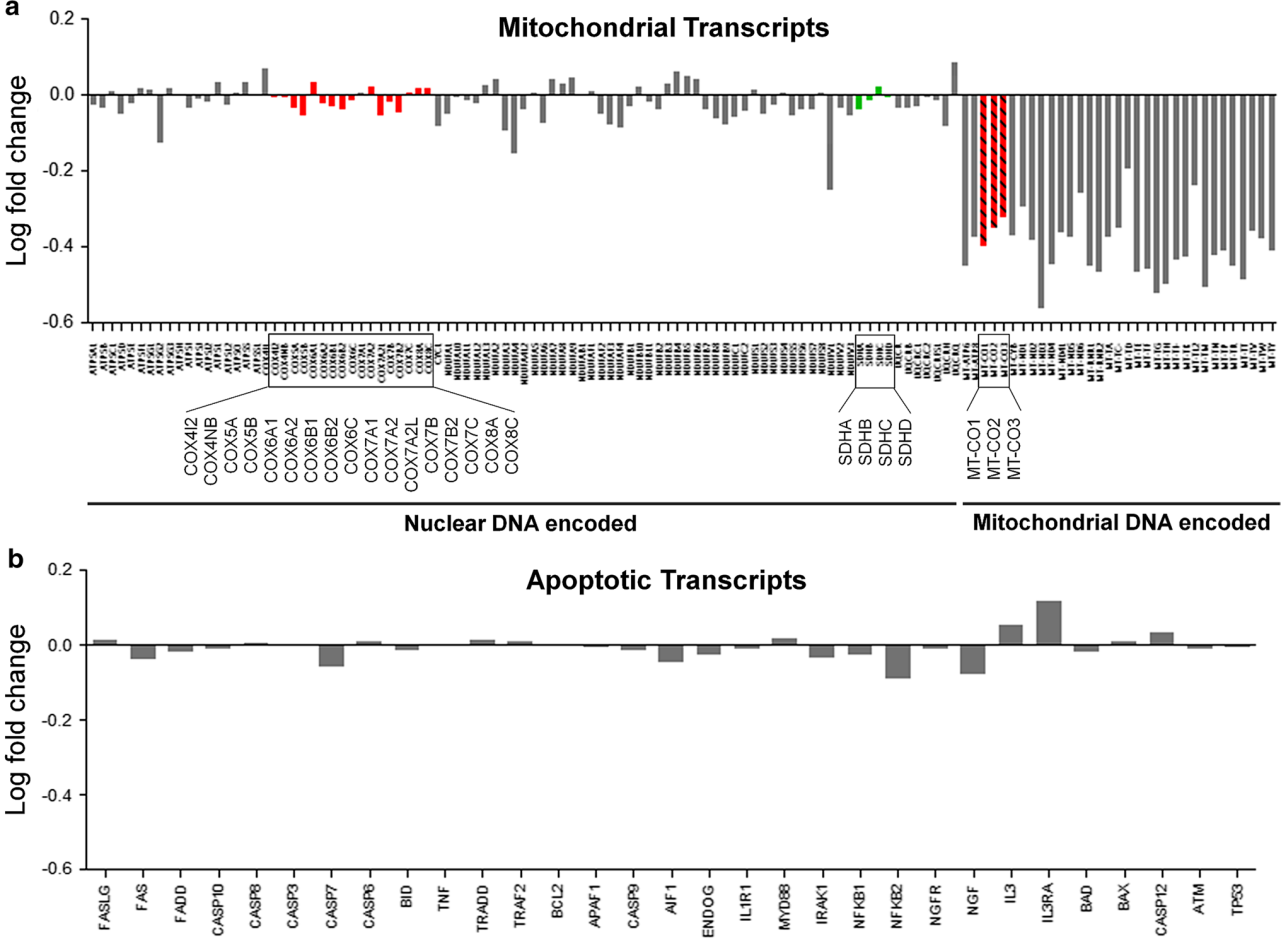
Relative abundance for mitochondrial and apoptotic transcripts in HSE. *Y* axis represents mean log (base 2) fold change in abundance for individual transcripts among HSE cases relative to controls. Replicate transcripts have been collapsed to their arithmetic mean. Negative values represent lower abundance in HSE tissue. **a** Mitochondrial DNA encoded transcripts exhibit lower abundance compared to nuclear DNA encoded mitochondrial transcripts (*right* compared to *left side* of *X* axis). Among transcripts corresponding to the cytochrome c oxidase enzyme complex, mitochondrial encoded transcripts (*red hatched bars*) exhibit lower abundance than nuclear encoded transcripts (*red solid bars*). For succinate dehydrogenase, nuclear encoded transcripts (*green solid bars*) exhibit relatively high abundance compared to mitochondrial encoded transcripts. **b** Transcripts encoding for apoptotic mediators did not show any consistent or significant changes in abundance in HSE tissue compared to controls

To aid comparison with control tissue, and to avoid using HSE tissue exhibiting frank necrosis, the gene-expression microarray experiments were undertaken using RNA recovered from the cingulate region. 287 transcripts exhibited significantly (FDR <5 %) lower abundance in HSE compared to control tissue (Fig. 2). MtDNA encoded transcripts represented only 398/62977 (0.6 %) of the total number of transcripts on the array (the array contained multiple probes for each mitochondrial gene). These mitochondrial transcripts were highly significantly over-represented, accounting for 219 out of 287 (76 %) transcripts in the significantly lower abundant set of transcripts (*p* < 0.0001). These transcripts encoded for 28 out of 33 MtDNA genes on the array (Tables S3 and S4; Online Resource 1). Examining all mitochondrial related transcripts (nuclear and mitochondrial genome encoded), there was a greater decline in abundance for MtDNA encoded compared to nuclear DNA encoded transcripts in HSE cases compared to controls. For example, focusing on cytochrome c oxidase, a mitochondrial enzyme composed of three mitochondrial encoded (CO1, CO2, CO3) and twelve nuclear encoded (COX4I1-COX8C) subunits, there was a lower abundance for mitochondrial compared to nuclear encoded transcripts in HSE cases (Fig. 3a).

In the set of transcripts exhibiting significantly lower abundance in HSE tissue, transcripts encoding for other cellular components were also significantly over-represented (smaller numbers of transcripts with weaker significance compared to mitochondrial components). These included the small ribosomal subunit, microtubule associated complex, kinesin complex, neuronal/dendritic spine and laminin (Table S5, Online Resource 1). No host transcripts exhibited significantly higher abundance in HSE compared to control tissue. Transcripts encoding for apoptotic mediators did not display any consistent changes in abundance in HSE tissue (Fig. 3b).

**Fig. 4:**
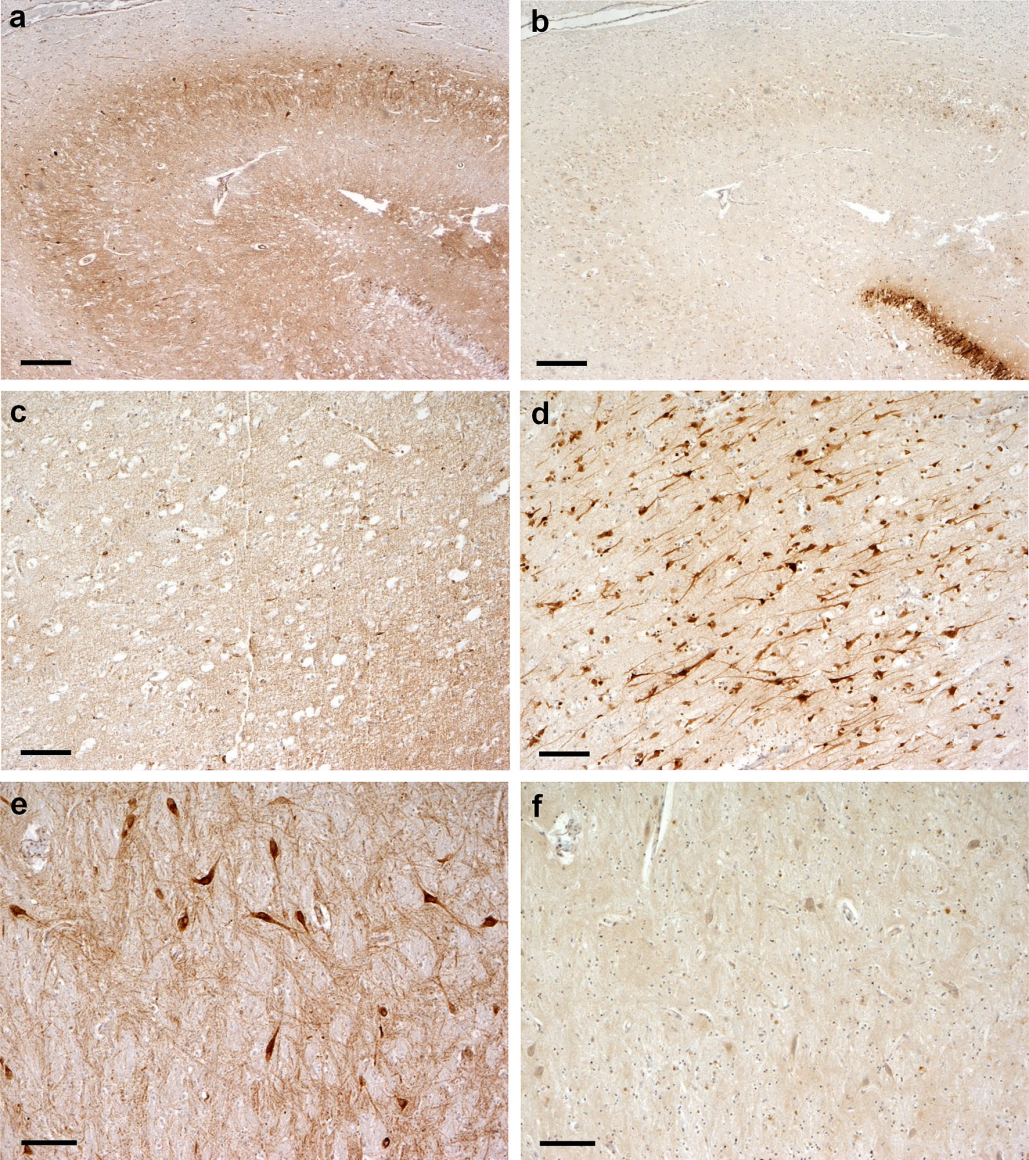
Pattern of distribution of CO1 and HSV-1 antigen expression in brain tissue from HSE patients. Consecutive brain tissue sections were immunoperoxidase stained for either CO1 or HSV-1 antigen. All regions show an inverse pattern of staining for CO1 and HSV-1. **a** Hippocampus, CA2 region, diffuse granular CO1 staining in the neuropil, with a relative lack of CO1 staining in the dentate gyrus. **b** Hippocampus, CA2 region (consecutive section); absence of HSV-1 staining in the neuropil, with strong HSV-1 staining restricted to the dentate gyrus. **c** Frontal cortex, moderate granular CO1 staining in neurons. **d** Frontal cortex (consecutive section); strong HSV-1 antigen staining in neurons. **e** Amygdala, strong CO1 reaction among large neurons. **f** Amygdala (consecutive section); limited HSV-1 antigen staining among large neurons. *Scale bars* 250 μm (**a**, **b**), 100 μm (**c**–**f**). Note; although strong HSV-1 antigen staining was observed in neurons, no intranuclear inclusions were observed in these cells

**Fig. 5:**
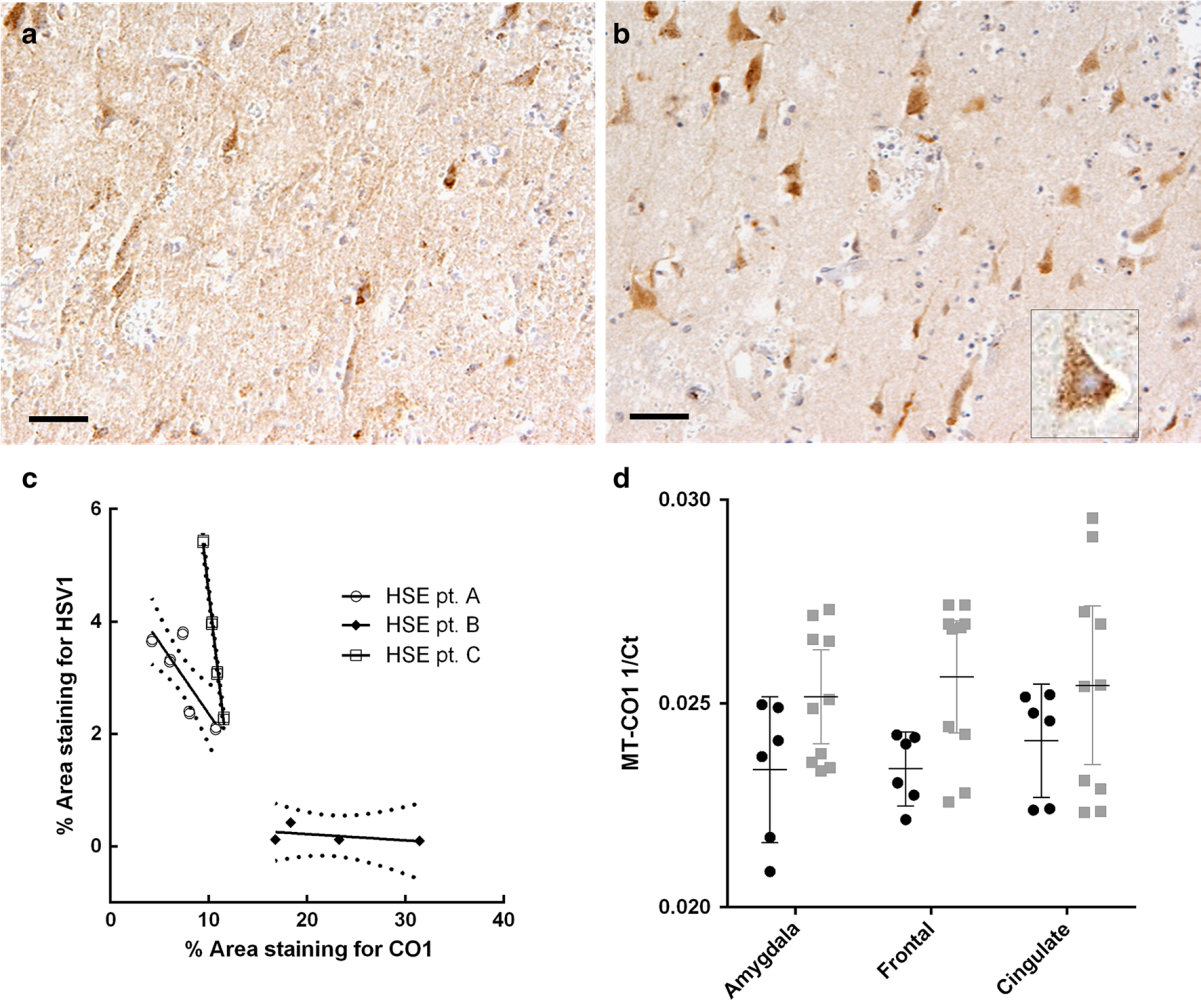
Quantification of CO1 and HSV-1 antigen expression in brain tissue from HSE patients. Consecutive brain tissue sections were immunoperoxidase stained for either CO1 or HSV-1 antigen. **a** Cingulate gyrus, moderate CO1 expression. **b** Cingulate gyrus, neurons with strong HSV-1 reaction. *Scale bars* 100 μm. **c** Quantification of immunostaining in consecutive sections of the cingulate cortex for two HSE cases (*A*, *C*); cingulate and insula for the third HSE case (*B*). The proportion (%) of the area of tissue staining positively for either CO1 or HSV-1antigen was measured in sets of ‘paired’ images of the same field of view [*n* = 4 images for case 1 (*squares*) and *n* = 5 for case 2 (*circles*)]. There is a significant negative correlation between HSV-1and CO1 antigen staining for two cases (*A*, *C*) with the third case (*B*) showing the same negative trend (Spearman rank correlation coefficient; *r* = −0.66, *p* = 0.044; *r* = −0.9, *p* = 0.005; *r* = −0.8, not significant; respectively, for HSE patients *A*, *C*, *B*). **d** Measurement of CO1 transcript abundance in tissue from amygdala, frontal and cingulate regions among HSE cases and controls. Abundance for CO1 is significantly lower in cases compared to controls (*p* = 0.032); 2-way repeated measures ANOVA over 3 brain regions (two tissue sections per region) for cases (*black circles*) against controls (*grey squares*). CO1 abundance measured as 1/*C*_T_ (PCR fractional cycle number for threshold fluorescence). Mean and 95 % CI presented [cases (*n* = 3) and controls (*n* = 5)]

Consecutive tissue sections across the different brain regions for HSE cases and controls were immunostained for CO1 and HSV-1. An inverse pattern of staining for CO1 and HSV-1 was observed within the HSE tissue (Figs. 4, 5a, b). The percentage of the tissue area stained for CO1 and HSV-1 was quantified in cingulate and insula regions (these regions exhibited limited necrosis) via Image J analysis software. There was an inverse correlation (Spearman rank correlation coefficient) between the percentage areas stained for HSV-1 and CO1 in all three patients; significant in two, the third patient showing the same trend (*r* = −0.66, *p* = 0.044; *r* = −0.9, *p* = 0.005; *r* = −0.8, not significant, respectively; Fig. 5c).

Interpretable results for dual IF and double IP staining were obtained from isolated cingulate sections in a single HSE patient. The remaining sections exhibited poor reactivity. An insufficient number of images could be obtained to undertake a systematic assessment of HSV and mitochondrial (CO1 or TOMM70) staining. Accepting this limitation, qualitatively, the IF staining captured a single HSV-1 infected cell which exhibited nuclear (DAPI) but not mitochondrial (TOMM70) stain. In contrast, cells in the nearby tissue stained for TOMM70. Similarly, IP staining of the cingulate tissue demonstrated widespread nuclear staining for HSV-1, with occasional dense cytoplasmic staining for CO1; Supplementary Figures S1 and S2 (Online resource 2).

Transcript abundance for CO1 and COX6A1 (mitochondrial and nuclear DNA encoded enzyme subunits for CO) were re-measured in brain tissue using qPCR (Fig. 5d). Two experimental replicates from each brain region (cingulate, amygdala, and frontal) were examined among HSE cases (*n* = 3) and controls (*n* = 5). Both CO1 and COX6A1 exhibited lower abundance in cases compared to controls across the brain regions. The reduction was only significant for CO1 [*p* = 0.032 (two-way repeated measures ANOVA)], with a greater reduction in abundance for CO1 than COX6A1 [mean *C*_T_ difference 3.63 ± 95 % confidence intervals (CI) 1.48–5.77 versus 1.57 ± −2.65–5.78 for CO1 and COX6A1, respectively].

**Fig. 6:**
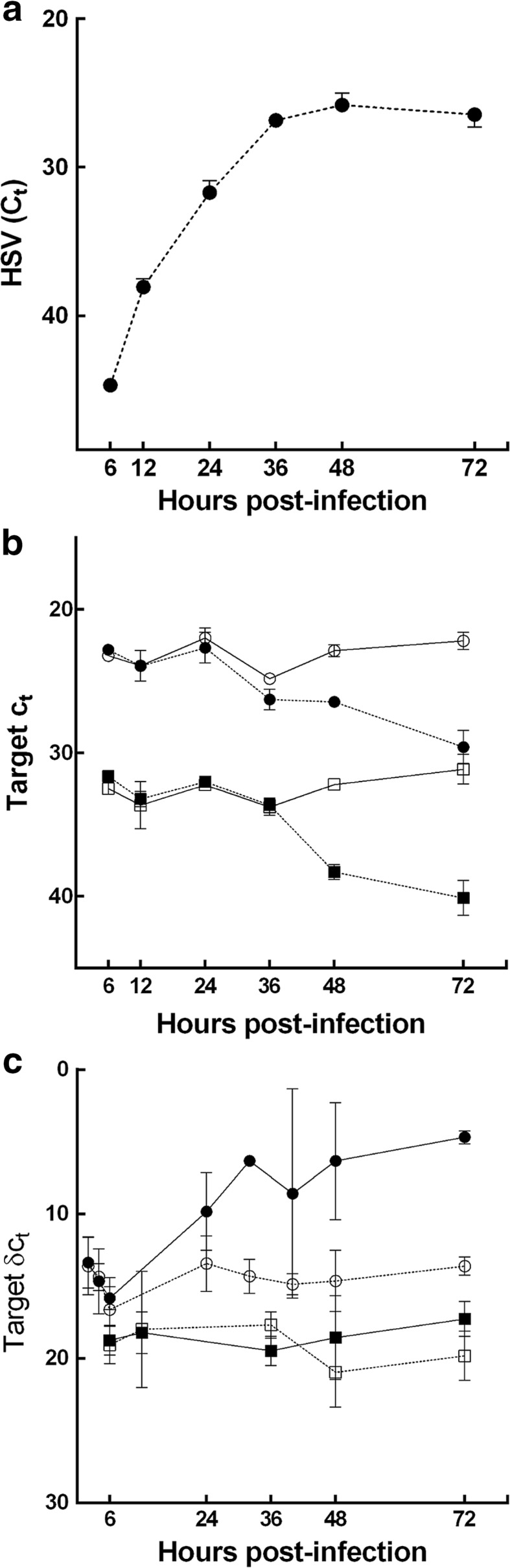
HSV-1 and host transcript abundance during in vitro HSV-1 infection. Viral DNA and host RNA from the infected human astrocytes were examined at indicated time-points via qPCR and qRT-PCR, respectively. **a** Increase in HSV-1 DNA (*solid circles*) abundance (*C*_T_) during the course of infection, reaching a maximum at 48 h pi. **b** Decrease in transcript abundance (*C*_T_) for CO1 (*circles*) and DAD1 (*squares*) among infected (*solid symbols*) relative to non-infected cells (*open symbols*), reaching a nadir at 72 h pi. Early decrease in CO1 (*solid circles*) over the first 36 h, with relative abundance [*δC*_T_ (CO1-DAD1)] significantly lower at 36 h pi (*p* = 0.036). At the same time-point, no change in DAD1 abundance between infected and non-infected cells. **c** TNF (*circles*) exhibits a significant increase in relative abundance [*δC*_T_ (TNF-DAD1)] at 72 h pi (*p* = 0.029) among infected (*solid circles*) relative to non-infected astrocytes (*open circles*). CASP3 (*squares*) exhibits no significant change in relative transcript abundance [*δC*_T_ (CASP3-DAD1)] during infection. Data presented as mean ± 95 % confidence interval (CI) for each experimental group (minimum of 3 replicate experiments per group). Differences in *C*_T_ at 36 and 72 h pi assessed using the Mann–Whitney *U* test

Next, we used cultures of primary human astrocytes to investigate the HSV-1 related mitochondria damage over time. Purity of the astrocyte cultures was confirmed via GFAP labelling. Active HSV-1 infection was confirmed by a high proportion of astrocytes exhibiting intranuclear inclusions [Figs. S3 and S4 (Online resource 3)]. During in vitro infection, HSV-1 DNA abundance increased, reaching a maximum at 48 h pi (Fig. 6a). There was a reduction in both mitochondrial (CO1) and nuclear (DAD1) transcripts during HSV-1 infection relative to non-infected cells. At 36 h pi, CO1 transcript abundance was lower among infected compared to non-infected cells. This early reduction was not observed for DAD1 (Fig. 6b). CO1 corrected for DAD1 abundance was significantly lower at this time-point (*p* = 0.036). TNF transcript abundance increased during infection relative to non-infected cells (Fig. 6c). The apoptotic mediators, BCL2, BAX, BAK, CASP3, CASP8, CASP9, PUMA, NOXA, and MCL-1 did not show any significant changes in transcript abundance during infection [Fig. 6c, Fig. S5 (Online resource 4)]. Non-infected astrocytes did not exhibit any significant changes in transcript abundance.

**Fig. 7:**
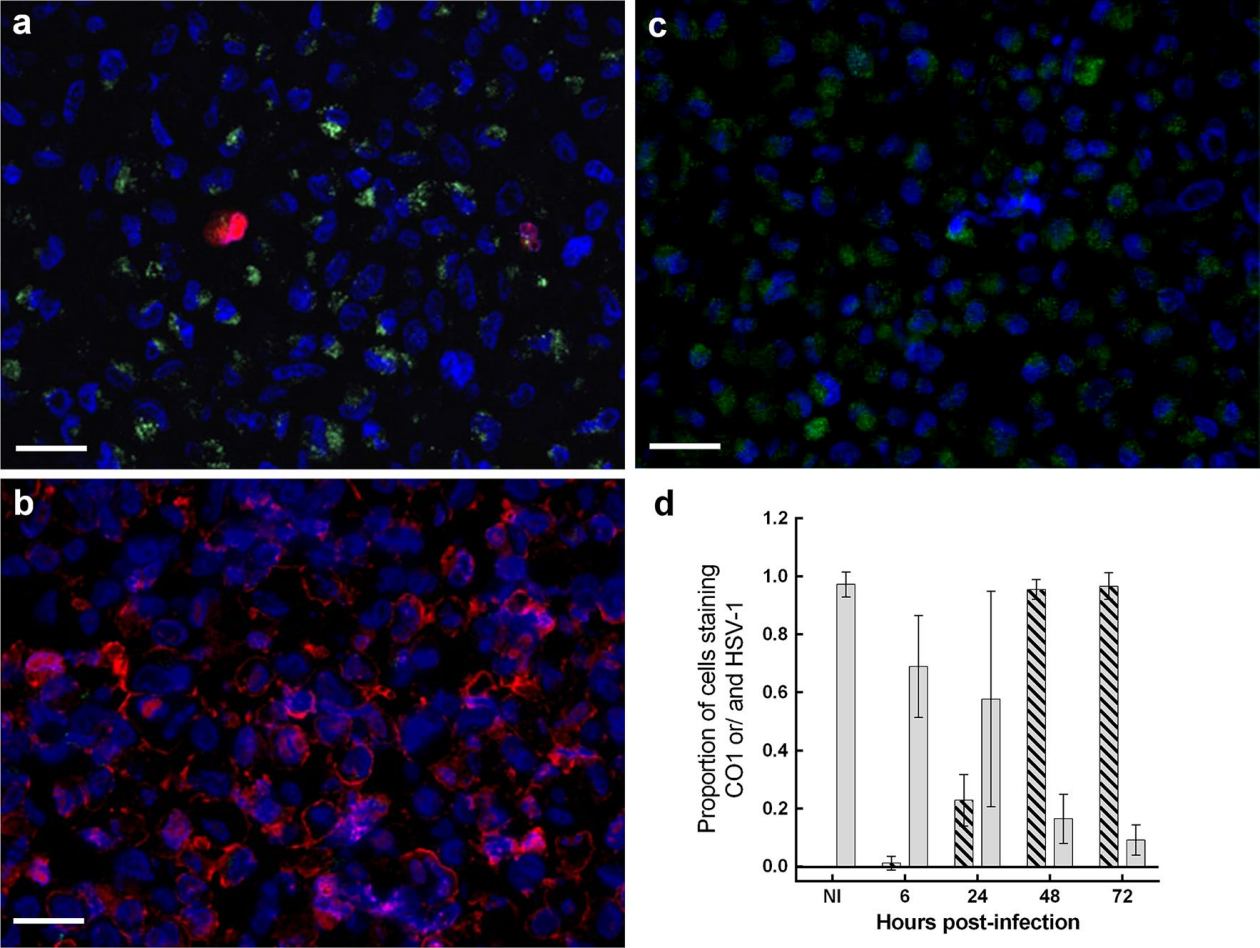
CO1 and HSV-1 antigen expression during in vitro HSV-1 infection. Dual immunofluorescence: infected human astrocytes were labelled CO1 (*green*) and HSV-1 (*red*) antigens with DAPI nuclear counter-stain (*blue*). **a** At 6 h pi, a granular reaction for CO1 is detected in majority of cells, with HSV-1 antigen expression observed in occasional cells. **b** At 72 h pi, HSV-1 antigen is expressed in majority of cells, with no apparent CO1-positive cells. The nuclei appear slightly larger compared to early infected and non-infected cells. This may represent nuclei swelling following infection [see TEM images (Fig. 10)]. **c** Non-infected astrocytes are positive for CO1, with no HSV-1 expression at 72 h pi. *Scale bars* 100 μm. **d** Plot of proportion of astrocytes expressing HSV-1 and/or CO1 antigen among non-infected (ni) and HSV-1 infected cultures at 6, 24, 48 and 72 h pi. *Y* axis—proportion. *X* axis—hours pi. *Grey bars* CO1 expressing cells. *Hatched bars* HSV-1 expressing cells. There is progressive reduction in CO1 and steady rise in HSV-1 antigen expression over time pi. Proportions are corrected for number of positive DAPI (nuclear) staining cells

Among astrocytes recovered from the infection time-course, there was progressive reduction in the proportion of DAPI positive cells expressing CO1, while the proportion expressing HSV-1 increased, over time pi. Non-infected cells exhibited stable CO1 expression, with no HSV-1 detected (Fig. 7).

**Fig. 8:**
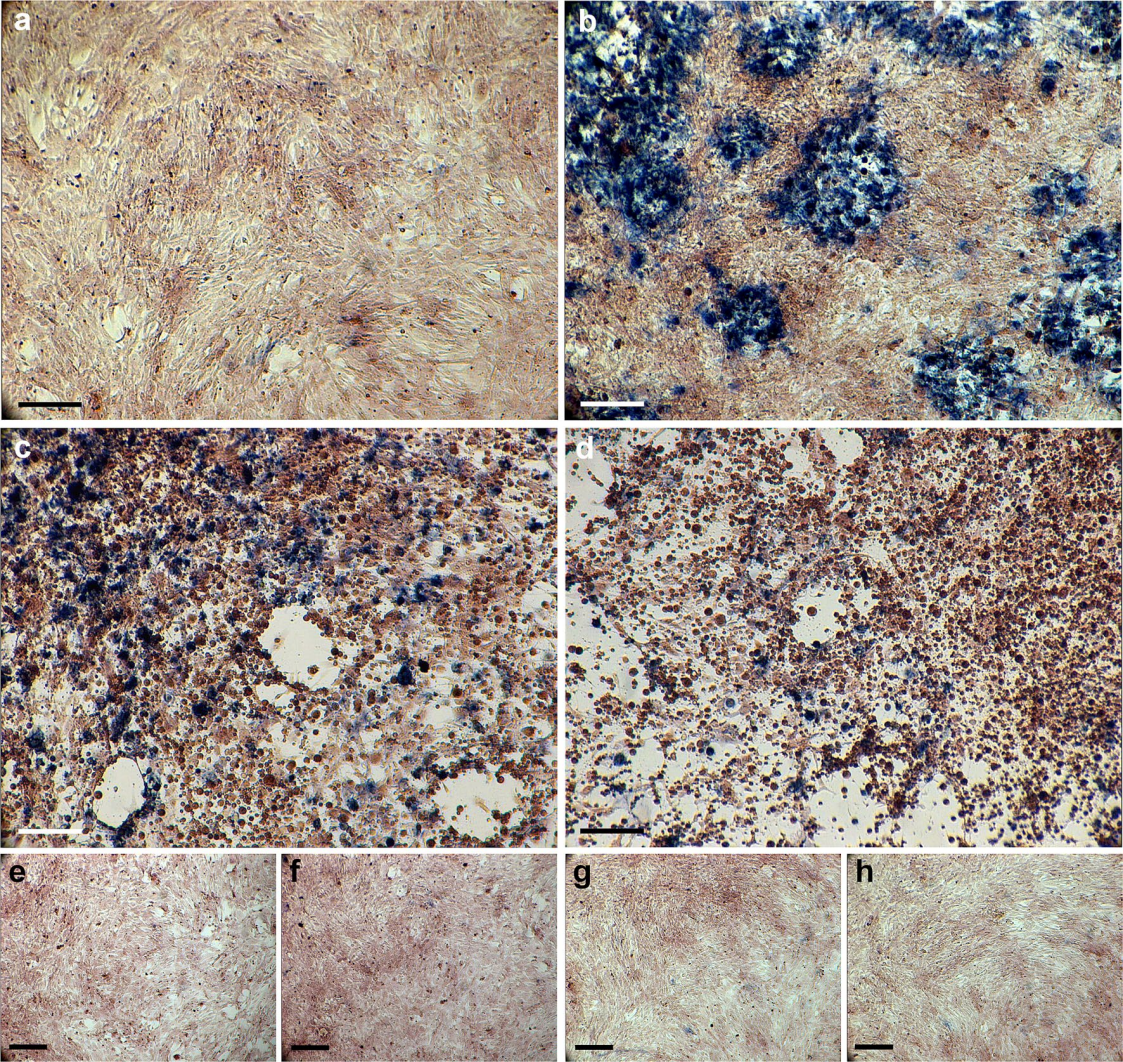
Mitochondrial enzyme function during in vitro HSV-1 infection. **a**–**d** Sequential enzyme histochemistry of cytochrome c oxidase CO (*brown*) and succinate dehydrogenase SDH (*blue*) mitochondrial enzyme activity demonstrate an early loss of CO function during infection. **a** At 6 h pi, uniform brown stain indicates that CO is functional. **b** At 24 h pi, early impairment of CO activity, with sustained SDH activity, is evidenced by patches of blue, CO-negative, SDH-positive cells. **c**, **d** At 48 and 72 h pi, both CO dysfunction and cell death is evidenced by further blue patches and loss of continuity of the cell monolayer. **e**–**h** In non-infected astrocytes, CO remained active throughout the time-course [6 h (**e**), 24 h (**f**), 48 h (**g**) and 72 h pi (**h**)]. *Scale bars* 200 μm (**a**–**d**), 300 μm (**e**–**h**)

**Fig. 9:**
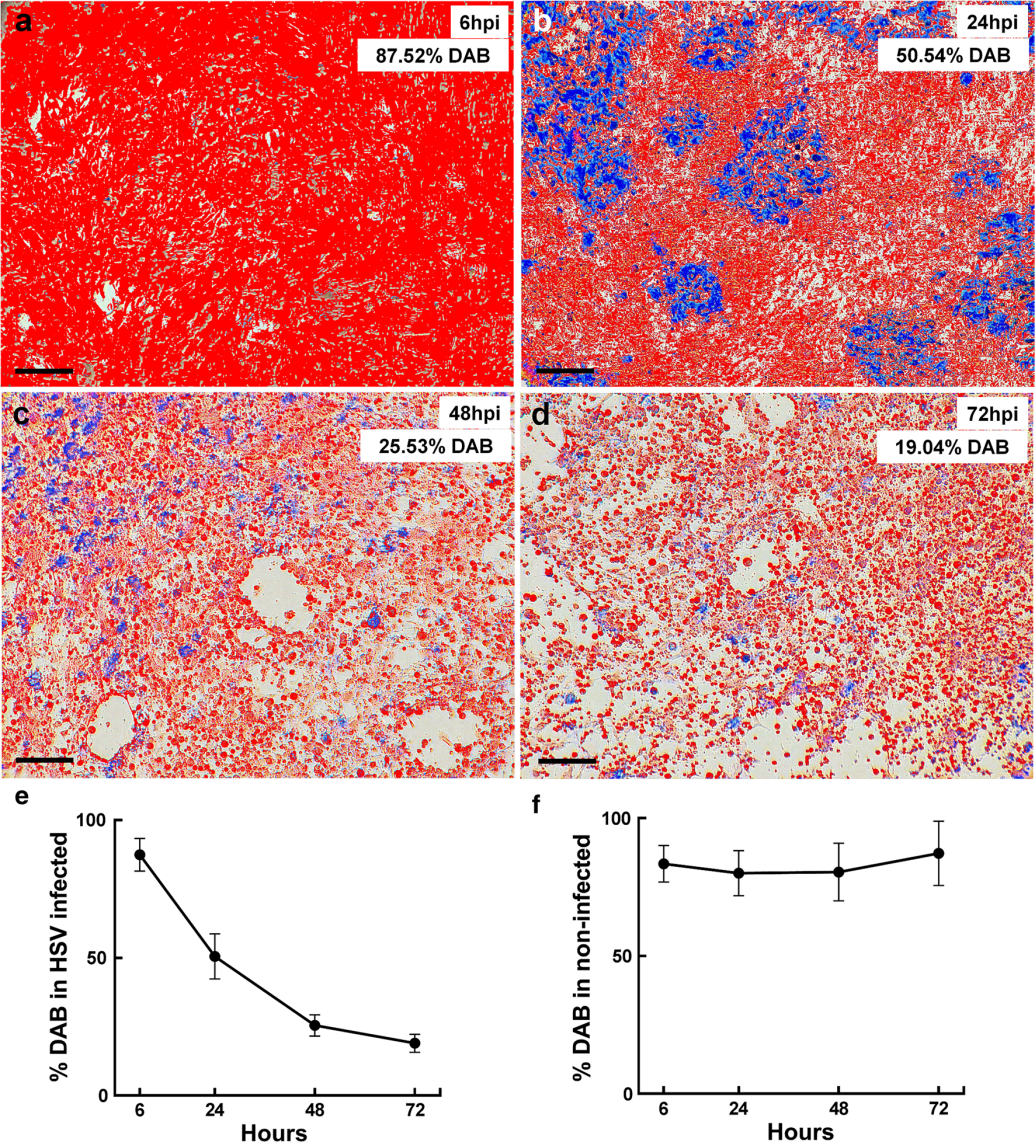
Quantification of cytochrome c oxidase enzyme function during in vitro HSV-1 infection. The proportion of cells adherent to the flask surface (within the monolayer) and exhibiting intact cytochrome c oxidase (CO) function was quantified. **a**–**d** The area (%) of the monolayer staining positively for DAB (*false red*) was measured at 6, 24, 48 and 72 h pi. **e** Area of DAB staining declined from 87.52 to 50.54, 25.53 and 19.04 % over time pi. **f** Area of DAB staining remained above 80 % throughout time-course among non-infected cells, data are presented as mean ± 95 % CI for each time-point. Replicate cultures were examined per time-point (*n* = 3)

Sequential histochemical testing for CO and then SDH activity in infected astrocytes was used to demonstrate an early loss of CO function. Like many mitochondrial enzymes, CO is composed of subunits encoded by both mitochondrial and nuclear genome. In contrast, SDH is the only enzyme in the respiratory chain composed of subunits encoded entirely by the nuclear genome. Sequential histochemistry is used as a functional screen for mitochondrial genome related defects in clinical settings [11]. If enzymes derived from the mitochondrial genome are selectively impaired during HSV-1 infection, a loss of CO function but maintenance of SDH function would be expected (Figs. 8, 9).

At 6 h pi, the majority of the monolayer stained with DAB, indicating intact CO function (Figs. 8a, 9a). At 24 h pi, multiple ‘blue patches’ were observed in the monolayer. These ‘blue patches’ represented cells with impaired CO function (failure to oxidise DAB), but maintenance of SDH function [sustained ability to reduce NBT (Figs. 8b, 9b)]. Later, further blue patches of cells and loss of cells from the monolayer were observed, with a progressive decrease in the area of DAB staining (Figs. 8c, d, 9c, d). Among non-infected astrocytes the majority of the monolayer stained with DAB throughout the time-course (Fig. 8e–h). Independent review of masked images gave consistent results; ranked non-infected, 6, 24, 48 and 72 h pi for decreasing area of DAB staining.

**Fig. 10:**
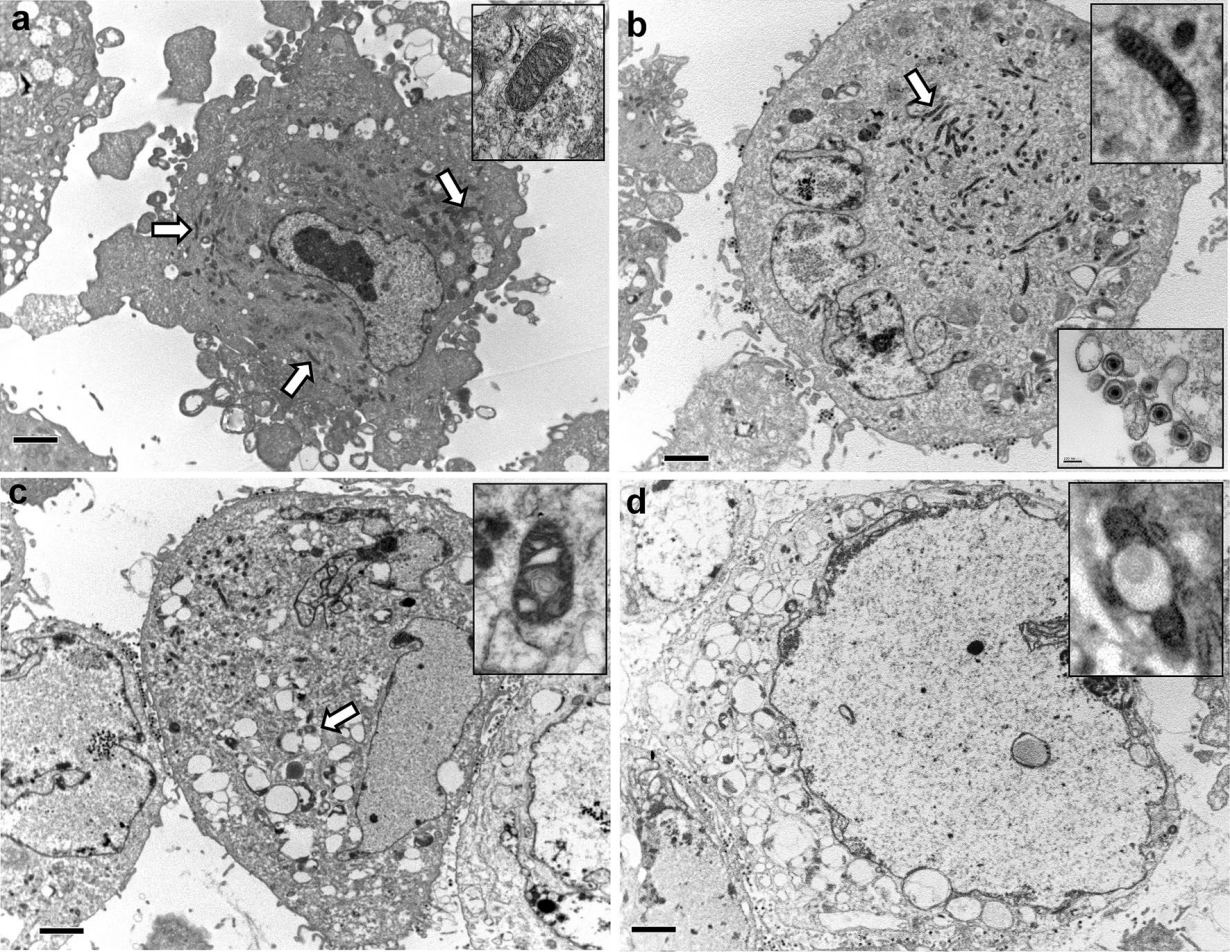
Ultra-structural cellular changes following in vitro HSV-1 infection. **a** In non-infected cells, mitochondria are scattered throughout the cytoplasm (*arrows*) with a centrally located nucleus exhibiting granular chromatin. **b** ‘Early’ morphological changes: HSV-1 infected cell exhibit central clustering in the cytoplasm (*arrow*) of ‘condensed’ mitochondria (*top inset*). Viral particles are detected in the nucleus, cytoplasm and budding on the surface of the cell (*bottom inset*). **c** ‘Intermediate’ morphological changes: mitochondria are swollen (*arrow*/*inset*) with intra-cristal enlargement and whirling of the mitochondrial membrane (*inset*). The nucleus exhibits loss of chromatin granularity. **d** ‘Late’ morphological changes: mitochondria are markedly enlarged (*inset*); the cell is dramatically swollen, the nucleus exhibits a severe loss of chromatin granularity and its membrane appears fragmented. *Scale bars* 2 μm

There was a progressive reduction in viable astrocyte cell number (unstained for trypan blue) among infected cultures over the time-course. Cell number in non-infected cultures remained stable. At 48 and 72 h pi, infected astrocytes changed from a stellate to a globoid morphology with loss of their astrocytic processes [Table S6 and Fig. S6 (Online resource 4)].

Transmission electron microscopy of cultured astrocytes demonstrated consistent ultra-structural changes within infected cells. These changes were classified as ‘early’, ‘intermediate’ and ‘late’. At 24 h pi, the majority of cells exhibited ‘early’ changes. At 72 h pi, the majority of cells exhibited ‘late’ changes. The ultra-structural changes were in agreement with previous reports [7, 36]. In the ‘early’ phase, mitochondria were clustered in the central cytoplasm. They appeared condensed with increased electron density of their cristae. No structural disarrangements of nuclear chromatin or membrane were observed (Fig. 10b). During the ‘intermediate’ phase, mitochondria were enlarged, with intra-cristal swelling and whirling of their membranes. Cells were swollen, with loss of chromatin granularity (Fig. 10c). In the ‘late’ phase, mitochondria were severely swollen and degraded. There was dramatic swelling of the cell and nucleus, loss of chromatin granularity, and membrane disintegration (Fig. 10d). Mature enveloped virions decorated the extracellular aspect of the plasma membrane at all time-points pi (Fig. 10b, inset). Non-infected astrocytes did not exhibit cellular degradation or HSV-1 particles (Fig. 10a). There was a steady decline in the number of normally conformed mitochondria within cells with time pi [mean 35.8 (±95 % CI 23.2–48.4); 17.6 (8.1–27.1); 13.4 (2.9–23.8); 3.2 (−1.9 to 8.3); for non-infected, 24, 48 and 72 h pi, respectively].

**Fig. 11:**
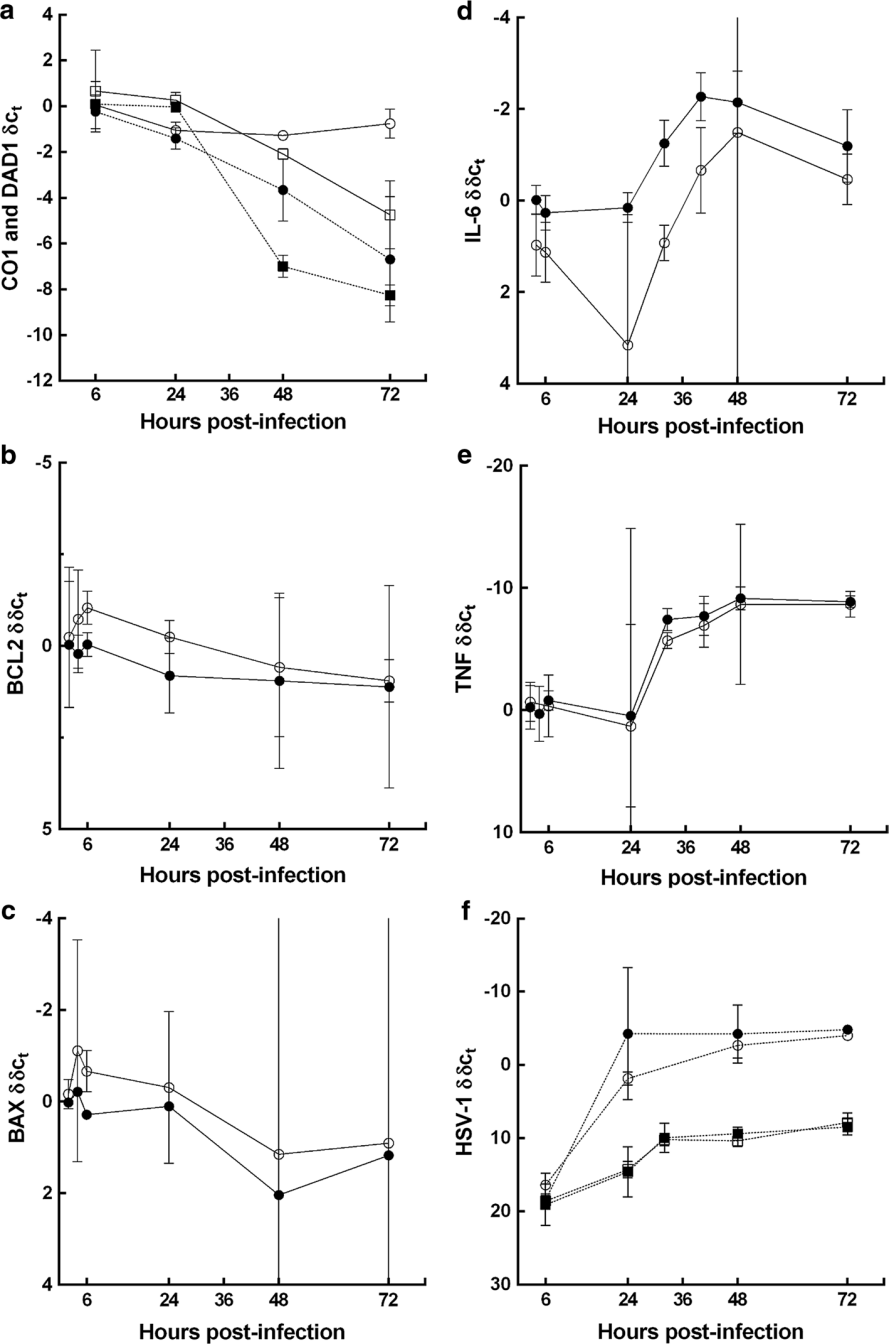
Comparison of host transcript and HSV-1 DNA abundance in minocycline treated and non-treated astrocytes during in vitro HSV-1 infection. Viral DNA and host RNA from infected human astrocytes were examined at indicated time-points via qPCR and qRT-PCR, respectively. Relative transcript abundance. **a** CO1 (*circles*) and DAD1 (*squares*), **b** BCL2, **c** BAX, **d** IL-6, **e** TNF in HSV-1 infected non-treated (*solid symbols*) compared to HSV-1 infected minocycline treated (*open symbols*) astrocyte cultures; **f** HSV-1 DNA in non-treated (*solid circles*) compared to minocycline (60 µM) treated (*open circles*); minocycline + aciclovir (20 µM) co-treatment (*open squares*) and aciclovir (20 µM) alone (*solid squares*) among HSV-1 infected astrocytes. *Y* axis—relative transcript abundance *δC*_T_ (difference in *C*_T_ in infected minus non-infected; **a** or *δδC*_T_ (difference for target gene—DAD1 in infected minus non-infected cultures; **b**–**f** *X* axis—hours pi. Data are presented as mean ± 95 % CI for each experimental group (minimum of 4 experiments per group). **a** CO1 exhibits sustained abundance during infection in minocycline treated compared to non-drug exposed cells. There is a significant difference in relative transcript abundance [*δδC*_T_ (CO1-DAD1)] between exposures (*p* = 0.029) at 48 h pi. DAD1 exhibits a smaller decrease in transcript abundance (*δC*_T_) in minocycline compared to non-drug exposed cells (not-significant). **b** BCL2 and **c** BAX exhibit significant increases in relative transcript abundance among minocycline exposed compared to non-drug exposed cultures between 2 and 24 h pi (*p* = 0.004, *p* = 0.012, respectively). **d** IL-6 exhibits significant decrease in transcript abundance among minocycline treated compared to non-drug exposed astrocyte cultures between 4–72 h pi (*p* < 0.0001). **e** TNF exhibits similar trend to IL-6. **f** HSV-1 DNA shows no significant change in relative transcript abundance [*δδC*_T_ (HSV-1 minus DAD1) in infected minus non-infected cultures] in either minocycline treated compared to non-treated or minocycline and acyclovir co-treated compared to aciclovir alone. Significant differences in *δδC*_T_ were assessed using 2-way repeated measures ANOVA (column factor—drug exposure; row factor—time pi) using time-points: 2, 4, 6, 24 h (**b**, **c**) and 4, 6,24, 32, 48, 72 h (**e**). The Mann–Whitney *U* test was used to compare corrected CO1 abundance at 48 h pi (**a**)

**Fig. 12:**
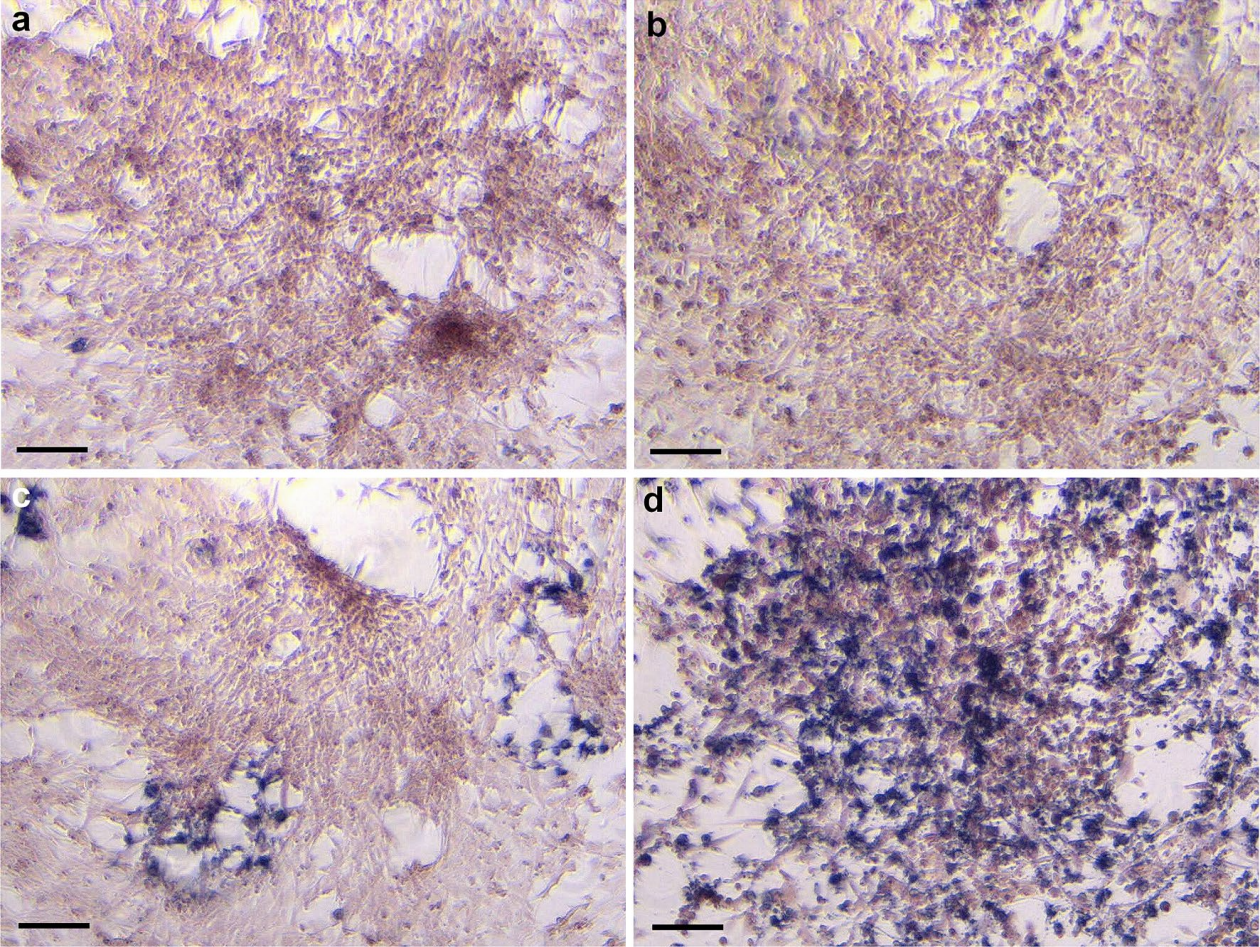
Comparison of cytochrome c oxidase function in minocycline treated and non-treated astrocytes during in vitro HSV-1 infection. Sequential enzyme histochemistry of cytochrome c oxidase CO (*brown*) and succinate dehydrogenase SDH (*blue*) activity demonstrates protective properties of minocycline. **a**, **b** At 24 and 48 h pi, vast majority of cells stain brown indicating intact CO function in minocycline treated cells. **c**, **d** At 24 and 48 h pi, obvious patches of cells stain blue, indicating impaired CO, but sustained SDH activity among non-treated cells. There is also a greater loss of the cell monolayer (reduced area stained for CO or SDH activity) among non-treated cells Area (proportions) for monolayer staining is documented in Table S7 (Online resource 5). *Scale bars* 200 μm

There was significantly higher CO1 transcript abundance in minocycline treated compared to non-treated infected astrocyte cultures [*p* = 0.029 (normalised for changes in DAD1); Fig. 11a]. Sequential histochemical testing demonstrated maintenance of CO activity at 24 and 48 h pi among minocycline treated infected cells (Fig. 12a, b). In contrast, prominent blue patches, indicating loss of CO but maintenance of SDH activity, were seen at 24 and 48 h pi among non-treated cells (Fig. 12c, d). At 48 h pi, there was also a significantly greater loss of the cell monolayer (reduced area stained for CO or SDH activity) among non-treated compared to treated cells [*p* = 0.046; area (proportion) of staining is documented in Table S7 (Online resource 5)].

Transcript abundances for BCL2 and BAX were significantly increased over 2–24 h pi in minocycline treated compared to non-treated infected astrocytes (*p* = 0.004, *p* = 0.012); IL-6 was significantly decreased over all time-points in treated cells (*p* < 0.0001). TNF also showed a trend for decreased abundance in treated cells (Fig. 11b–e).

There was no significant difference in HSV-1 DNA abundance in minocycline and aciclovir co-treated cultures, compared to those treated with aciclovir alone during infection (Fig. 11f). To confirm whether minocycline had anti-viral properties, we examined HSV-1 replication in the presence and absence of the drug in HeLa cells. The results indicated minocycline alone promoted viral replication. However, there was no significant difference in HSV-1 replication in aciclovir and minocycline co-treated cells compared to aciclovir alone (Online resource 5). Exposing uninfected astrocytes to minocycline, there was no change in cell number, morphology or viability compared to non-exposed cells over 72 h (Figs. S6, S9 and S10; Online resource 4 and 5).

## Discussion

This is the first investigation of the human host transcriptome in HSE brains and the first study of the effect of minocycline on HSV-1 infection.

Host transcriptome analysis demonstrated a profound reduction in mitochondrial related transcript abundance in post-mortem HSE brain tissue. Transcripts encoded by MtDNA were significantly over-represented among low abundant host transcripts. Reductions in mitochondrial transcript abundance were not observed in control tissue, indicating the changes were not secondary to agonal or post-mortem processes. Our human brain tissue findings support previous studies in non-neuronal cell lines, demonstrating a preferential and marked decline in mitochondrial transcripts and MtDNA following HSV infection [33, 45]. While some degree of MtDNA damage has been linked to many degenerative and/or pathological processes, profound MtDNA depletion is less common. The latter process has been reported in association with HSV-1, HSV-2 and EBV virus infection. It has also been reported in genetic mitochondrial syndromes, profound oxidant exposure and zidovudine therapy [9, 18, 43, 49].

Beyond mitochondrial related transcripts, we observed a decline in transcripts encoding for the small ribosomal subunit, the microtubule associated complex; neuronal/dendritic spine, kinesin complex and laminin. It is well reported, HSV interacts with small and large ribosomal subunits during active infection and subverts control of neuron axonal transport (including the kinesin complex), microtubule movement, and neuronal dendrite formation during infection [14, 16, 17, 30, 35]. Susceptibility of cells to HSV-1 infection is reported to vary depending on the extracellular matrix [48]. Reduced transcript abundance for laminin, a component of extracellular matrices, may again be linked with HSV-1 infection. We also observed a general reduction in host nuclear transcript abundance in HSE tissue compared to controls. Again, this supports previous in vitro studies reporting viral host shut-off of transcription and a decrease of cellular RNA abundance following HSV infection [31, 33, 34].

Supporting the decrease in mitochondrial transcripts, there was a corresponding reduction in CO1 protein in HSE tissue. CO1 staining was reduced in neurons and supporting cells, including astrocytes. Furthermore, there was an inverse correlation between CO1 and HSV-1 expression within the HSE cases. Tissue regions with high HSV-1 expression exhibited low CO1 expression and vice versa. Our brain tissue findings, support previous studies in non-neuronal cell lines demonstrating a reduction in CO protein following HSV infection [40]. In addition, the inverse correlation between CO1 and HSV-1 expression supports previous in vitro studies in non-neuronal cell lines indicating mitochondrial damage is mediated by active HSV-1 infection [45]. Astrocytes are increasingly recognized to have a pivotal role in protecting and supporting neuronal function [2]. Consequently, mitochondrial damage, both directly within neurons, and indirectly within astrocytes, has potential to contribute to neuronal injury during HSE.

We undertook in vitro studies to explore the temporal relationship between HSV infection and the mitochondrial changes observed in HSE tissue. There was a steady decline in CO1 transcript and protein abundance as HSV-1 infection progressed. We observed a progressive reduction in both CO1 (MtDNA encoded) and DAD1 (nuclear encoded) transcripts. However, there was an earlier reduction in transcript abundance in CO1 compared to DAD1. Similarly, we observed a progressive reduction in CO1 antigen staining, with an increase in HSV-1 staining, among DAPI positive astrocytes during infection. Infected astrocytes exhibiting impaired CO enzyme activity at 24 h pi were still firmly attached to the flask surface and retained their stellate morphology. Similarly, cell ultra-structural changes such as central migration of mitochondria and cristae condensation were observed prior to changes in the nucleus. Saffran and colleagues previously demonstrated a rapid and preferential loss of mitochondrial transcripts following HSV-1 in vitro infection of non-neuronal cells [45]. Migration of HSV-1 proteins into mitochondria has been reported to trigger degradation of MtDNA, i.e. via pUL12.5, or suppress mitochondrial enzyme activity, i.e. via US3 [10, 13, 45]. Our in vitro findings, together with these reports, support the notion that mitochondrial dysfunction is an early process during HSV-1 infection, which occurs before nuclear and cellular functions are significantly impaired.

This is the first study to demonstrate an early loss of mitochondrial function among respiratory chain enzymes encoded by MtDNA following HSV-1 infection. CO is the last enzyme in the electron transport chain (complex IV). CO is composed of enzyme subunits encoded via both MtDNA (i.e. CO1, CO2 and CO3) and nuclear DNA (e.g. COX6A1). In contrast, SDH is a respiratory chain enzyme (complex II) whose subunits (i.e. SDH A, B, C and D) are entirely encoded by nuclear DNA [11]. Infected astrocytes demonstrated impaired CO function at 24 h pi. In contrast, SDH function was maintained at 24 h. This early loss of CO function, but maintenance of SDH function, could be explained by loss of MtDNA encoded transcripts. CO1, CO2 and CO3 all exhibited low transcript abundance, whereas SDH A-D showed relatively higher abundance in HSE tissue. HSV-1 protein, pUL12.5, is reported to migrate into mitochondria during infection, and trigger a rapid degradation of MtDNA within 48 h pi, via the viral product’s exonuclease activity [10]. Indirect mechanisms could also trigger MtDNA depletion during HSV-1 infection, such as increased oxidant stress or viral induced alterations (transcriptional and/or post-transcriptional) in host mitochondrial repair, programmed cell death or transcriptional pathways [34, 44, 49].

HSE has classically been described as a necrotizing process [5, 21]. More recently, apoptosis has also been observed to contribute to death of neurons and glia in HSE brain tissue [4, 12]. Some in vitro and in vivo models of HSE have reported brain cell death via apoptosis [3, 23], whereas other studies have reported an absence of apoptosis following in vivo HSV-1 brain infection [20]. During our in vitro studies, we observed changes in cell ultra-structure classically associated with necrosis, such as mitochondrial swelling, nuclear swelling, cell swelling and membrane disintegration [56]. There was no consistent up-regulation of transcript abundances for apoptotic mediators among in vitro infected astrocytes or HSE brain tissue. Again, these results suggest apoptosis is not the main mechanism of cell loss during HSE. While we accept consistent changes in host apoptotic pathways may be obscured by variable rates of cell death occurring among different cells in the brain tissue, we did observe a consistent reduction in mitochondrial related transcripts in HSE tissue, weakening the latter argument.

Minocycline is reported to limit brain cell damage in models of viral encephalitis, including West Nile virus, Japanese Encephalitis Virus, Reovirus and HIV. However, it exacerbated brain damage in models of Rabies virus [26, 38, 39, 42, 55]. To date, minocycline has not been studied in HSE. Minocycline is reported to sustain mitochondrial and brain cell function by several mechanisms, including inhibition of apoptosis, via induction of the mitochondrial anti-apoptotic mediator BCL-2, suppression of mitochondrial pro-apoptotic mediators (e.g. BAX), maintenance of mitochondrial membrane integrity, reduced leakage of cytochrome c into the cytoplasm and suppression of pro-inflammatory mediators [27, 53]. Our results indicate minocycline protects mitochondrial function and cell viability during HSV-1 infection, with reduced loss of CO1 transcripts, sustained CO activity and better maintenance of the cell monolayer. As previously reported, our findings suggest minocycline protects cells through multiple mechanisms [8, 51]. The pro-inflammatory mediators IL-6 and TNF exhibited decreased abundance, whereas the mitochondrial anti- and pro-apototic mediators, BCL-2 and BAX, exhibited increased abundance.

Minocyline is reported to reduce viral replication in HIV, West Nile, and JE viruses [38, 39, 55]. In astrocytes, minocycline did not impact on HSV-1 DNA abundance. In Hela cells, minocycline, alone, promoted HSV-1 replication. However, there was no change in virus replication with minocycline and aciclovir co-treatment. Further studies are required to explore minocycline’s mechanisms of action. Validation of minocycline’s mitochondrial protection will benefit from further work in human neurons and/or animal models of HSE.

The in vitro culture of primary human astrocytes may not reflect the complexity of HSV-1 infection during encephalitis. However, both human brain tissue and human astrocyte cultures exhibited astrocytic inclusion bodies (pathognomonic feature of human HSV-1 infection [21]). Both exhibited a selective loss of mitochondrial transcripts and/or mitochondrial protein that was linked to increasing abundance of HSV-1. Neither brain tissue nor astrocyte culture exhibited any consistent changes in abundance for apoptotic mediators following HSV-1 infection. Consequently, astrocyte cultures reflected key changes in human brain tissue from HSE cases.

In conclusion, HSE patients demonstrate a preferential loss of mitochondrial transcripts and a corresponding loss of mitochondrial protein in post-mortem brain tissue. In vitro studies showed early transcript reduction, early mitochondrial enzyme dysfunction and early mitochondrial ultra-structural damage, prior to nuclear and cell damage, following HSV-1 infection. Minocycline sustains mitochondrial transcript abundance, mitochondrial enzyme function and astrocyte viability. The study provides compelling data to stimulate further investigations of minocycline and other agents focussing on mitochondrial protection as an adjunctive treatment for HSE.

## Electronic supplementary material

Below is the link to the electronic supplementary material.

Supplementary material 1 (PDF 329 kb)

Supplementary material 2 (PDF 305 kb)

Supplementary material 3 (PDF 281 kb)

Supplementary material 4 (PDF 384 kb)

Supplementary material 5 (PDF 240 kb)
