# Supplementary material for: Herpes simplex encephalitis is linked with selective mitochondrial damage; a post-mortem and in vitro study
Source: Acta Neuropathol. 2016 Jul 25;132:433–51. doi: 10.1007/s00401-016-1597-2 (PMC4992034; doi:10.1007/s00401-016-1597-2)
Supplement: Supplementary file 1 — Supplementary material 1 (PDF 329 kb) [file 401_2016_1597_MOESM1_ESM.pdf]

File: ESM_1 (Online Resource 1)

Article Title: Herpes Simplex encephalitis is linked with selective mitochondrial damage; a post-mortem and in-vitro study.

Journal Name: Acta Neuropathologica

Authors: Małgorzata Wnęk, Lorenzo Ressel, Emanuele Ricci, Carmen Martinez, Julio Cesar Villalvazo Guerrero, Zarini Ismail, Patrick F. Chinnery, Colin Smith, Anja Kipar, Beate Sodeik, Tom Solomon, Michael J. Griffiths

Corresponding Author: Dr Michael J. Griffiths; e-mail: griffmj@liverpool.ac.uk; tel: +44-151-795-9657; fax: +44-151-795-5529

Table S1 Patient information

Patient	Age (yrs)	Sex	PMI (hrs)	Duration of illness/cause of death
HSE	58	M	48	9 days illness
HSE	53	M	60	3 weeks illness
HSE	81	F	N/A	7 days illness
Control	70	M	48	Head injury 2 months before death
Control	77	M	54	Pneumonia and myocardial infarction
Control	64	M	>48	Myocardial infarction
Control	80	F	24	Bronchopneumonia
Control	89	F	27	Bronchopneumonia

PMI (post-mortem interval) – time from death to post-mortem

Table S2 Summary results for pathological examination of Herpes simplex virus encephalitis cases

**CaseID**	**Anatomic regions**					
	Frontal cortex	Temporal cortex	Hippocampus	Cingulate gyrus	Insula	Amygdala
B3918 - A	+	+	+	+	na	++
B5125 - B	+++	+++	+++	++	++	+++
B5614 - C	+++	+++*	+++	+	++	++

Legend:

+ Viral Inclusions, neuronal necrosis and small perivascular cuffs are observed

++ In addition to above, marked gliosis, leukocytoclastic vasculitis (fibrinoid necrosis of vessels) and diffuse perivascular cuffs with lymphocyte infiltration of adjacent parenchyma

+++ As above but multiple large focal areas of haemorrhage and parenchymal rarefation/cavitation

* - numerous astrocytic inclusion bodies observed; na: not available

Table S3 Mitochondrial and nuclear genes down-regulated in HSE cases versus controls

Mitochondrial genes (n = 28)				Nuclear genes (n = 37)			
Gene	Fold chg.	Gene	Fold chg.	Gene	Fold chg.	Gene	Fold chg.
MT-TP	-214.3	MT-ATP6	-4.3	NRGN	-80.8	A_23_P406928	-3.2
MT-TK	-130.0	MT-TF	-4.0	MT3	-42.1	AGAP2	-3.1
MT-TC	-128.6	MT-TG	-3.9	MAP1A	-12.7	LOC100288578	-3.0
MT-TH	-84.8	MT-RNR1	-3.8	PEA15	-11.6	KIF1A	-3.0
MT-TT	-32.8	MT-RNR2	-3.6	RPS3	-10.1	BX095413	-2.7
MT-TM	-23.0	MT-CO1	-3.5	GDI1	-9.6	PALM	-2.6
MT-TE	-16.0	MT-ND2	-3.3	RNA28S5	-9.4	NDRG2	-2.4
MT-TI	-14.4	MT-TY	-3.2	FLJ44606	-9.0	RPS28	-2.3
MT-TW	-10.5	MT-CO3	-3.1	IDS	-7.5	AK129645	-2.2
MT-ND4	-10.5	MT-ND3	-3.0	ILDR1	-5.4	TM9SF4	-2.2
MT-TA	-7.2	MT-ND6	-2.9	ZNF2	-5.3	ES309389	-2.2
MT-TR	-6.7	MT-ND5	-2.8	HSCB	-5.2	A_23_P317056	-2.1
MT-ATP8	-5.7	MT-CYB	-2.5	MAOA	-4.8	MTND4P12	-2.0
MT-TN	-4.9	MT-CO2	-1.9	NCRNA00087	-4.6	LOC100288418	-1.7
				ENST00000454092	-4.5	EPB49	-1.6
				DDX6	-3.7	KIF1C	-1.2
				LAMA5-003	-3.6	CALM3	-1.0
				DACT3	-3.5	RNA18S5	-0.8
				A_24_P182122	-3.2		

All transcripts exhibited significantly lower abundance among HSE cases (n=3) compared to controls (n=5) (FDR<5%). Fold change of replicate transcripts in the set were collapsed to their arithmetic mean.

Table S4 Mitochondria-related categories over-represented with transcripts significantly less abundant in HSE cases

**Gene Onotology / Pathway**	**Small molecule metabolic process**	**Respiratory electron transport chain**	**Mitochondrion**	**Oxidation-reduction process**	**Mitochondrial respiratory chain complex I**	**Mitochondrial electron transport, cyt c to oxygen**	**Response to oxidative stress**	**ATP synthesis coupled electron transport**	**ATP synthesis coupled proton transport**
**Genes in differential dataset**	13	11	10	7	5	3	3	3	2
**Genes in InnateDb**	1283	103	1408	495	47	5	124	7	20
**P raw**	0.000	0.000	0.000	0.000	0.000	0.000	0.001	0.000	0.001
**P corrected**	0.000	0.000	0.003	0.001	0.000	0.000	0.019	0.000	0.009
**Genes**	IDS<br>MAOA<br>MT-ATP6<br>MT-ATP8<br>MT-CO1<br>MT-CO2<br>MT-CO3<br>MT-CYB<br>MT-ND2<br>MT-ND3<br>MT-ND4<br>MT-ND5<br>MT-ND6	MT-ATP6<br>MT-ATP8<br>MT-CO1<br>MT-CO2<br>MT-CO3<br>MT-CYB<br>MT-ND2<br>MT-ND3<br>MT-ND4<br>MT-ND5<br>MT-ND6	AGAP2<br>C5orf63<br>MAOA<br>MT-ATP6<br>MT-ATP8<br>MT-CO2<br>MT-CYB<br>MT-ND3<br>MT-ND4<br>MT-ND6	MAOA<br>MT-CO1<br>MT-ND2<br>MT-ND3<br>MT-ND4<br>MT-ND5<br>MT-ND6	MT-ND2<br>MT-ND3<br>MT-ND4<br>MT-ND5<br>MT-ND6	MT-CO1<br>MT-CO2<br>MT-CO3	MT-CO1<br>MT-ND3<br>MT3	MT-ND2<br>MT-ND4<br>MT-ND5	MT-ATP6<br>MT-ATP8

Table S5 Cellular components significantly over-represented among the set of low abundance transcripts

Cellular component	GO Id	Uploaded genes	Genes in IDB	p-value	Genes/Transcripts
mitochondrion	GO:0005739	14	1411	2.11E-06	AGAP2; C5orf63; HSCB; MAOA; MT-ATP6; MT-CO1; MT-CO2; MT-CO3; MT-CYB; MT-ND1; MT-ND2; MT-ND3; MT-ND4; MT-ND6
mitochondrial inner membrane	GO:0005743	12	353	6.08E-11	MT-ATP6; MT-ATP8; MT-CO1; MT-CO2; MT-CO3; MT-CYB; MT-ND1; MT-ND2; MT-ND3; MT-ND4; MT-ND5; MT-ND6
respiratory chain complex IV	GO:0045277	3	3	3.52E-07	MT-CO1; MT-CO2; MT-CO3
mitochondrial respiratory chain complex I	GO:0005747	5	47	1.10E-06	MT-ND1; MT-ND2; MT-ND3; MT-ND4;MT-ND5
mitochondrial proton-transporting ATP synthase complex	GO:0005753	2	21	0.0119	MT-ATP6; MT-ATP8
mitochondrial respiratory chain complex IV	GO:0005751	1	6	0.04587	MT-CO1
proton-transporting ATP synthase complex, coupling factor F(o)	GO:0045263	1	6	0.04587	MT-ATP6
mitochondrial proton-transporting ATP synthase complex, coupling factor F(o)	GO:0000276	1	7	0.04794	MT-ATP8
mitochondrial respiratory chain complex III	GO:0005750	1	7	0.04794	MT-CYB
small ribosomal subunit	GO:0015935	2	26	0.01735	RPS28; RPS3
cytosolic small ribosomal subunit	GO:0022627	2	41	0.02639	RPS28; RPS3
neuron spine	GO:0044309	1	4	0.03917	PALM
dendritic spine membrane	GO:0032591	1	7	0.04794	PALM
kinesin complex	GO:0005871	2	51	0.03225	KIF1A; KIF1C
microtubule associated complex	GO:0005875	2	33	0.02531	MAP1A; PEA15
laminin-10 complex	GO:0043259	1	3	0.03384	LAMA5
laminin-11 complex	GO:0043260	1	3	0.03384	LAMA5
laminin-5 complex	GO:0005610	1	4	0.03917	LAMA5
laminin-1 complex	GO:0005606	1	7	0.04794	LAMA5

Legend: GO;Id-cellular component defined by Gene Ontology database (GO; http://geneontology.org); Uploaded genes – number (no.) of genes uploaded from set of low abundance transcripts; Genes in IDB – no. of genes for Go Id present in InnateDB database (http://www.innatedb.com); P-value for over-representation after Benjamini Hochberg correction.

Primer references:

TNF (Life Technologies, TaqMan Gene Expression assay ID: Hs00174128_m1); CO1 (TaqMan: Hs02596864_g1); COX6A1 (TaqMan: Hs01924685_g1); CASP3 (TaqMan: Hs00234387_m1); DAD1 (TaqMan: Hs02912874_m1); HSV-1 F-5'-gcagtttacgtacaaccacatacagc, R-5'-agcttgcgggcctcgtt, Probe-5'-[6FAM]-cggcccaacatatcgttgacatggc-[TAM]; CASP8 (TaqMan: Hs01018151_m1); CASP9 (ID: Hs00154260_m1); BAX (ID: Hs00180269_m1); BAK1 (ID: Hs00832876_g1); BCL2 (ID: Hs00608023_m1); PUMA (ID: Hs00248075_m1); NOXA (ID: Hs00560402_m1); MCL1-1 (ID: Hs01050896_m1); IL-6 (ID: Hs00985639_m1)
