# Supplementary material for: Herpes simplex encephalitis is linked with selective mitochondrial damage; a post-mortem and in vitro study
Source: Acta Neuropathol. 2016 Jul 25;132:433–51. doi: 10.1007/s00401-016-1597-2 (PMC4992034; doi:10.1007/s00401-016-1597-2)
Supplement: Supplementary file 2 — Supplementary material 2 (PDF 305 kb) [file 401_2016_1597_MOESM2_ESM.pdf]

File: ESM_2 (Online Resource 2)

Article Title: Herpes Simplex encephalitis is linked with selective mitochondrial damage; a post-mortem and in-vitro study.

Journal Name: Acta Neuropathologica

Authors: Małgorzata Wnęk, Lorenzo Ressel, Emanuele Ricci, Carmen Martinez, Julio Cesar Villalvazo Guerrero, Zarini Ismail, Patrick F. Chinnery, Colin Smith, Anja Kipar, Beate Sodeik, Tom Solomon, Michael J. Griffiths

Corresponding Author: Dr Michael J. Griffiths; e-mail: griffmj@liverpool.ac.uk; tel: +44-151-795-9657; fax: +44-151-795-5529

Figure S1 Immunofluorescent image of HSV-1 and TOMM70 in HSE patient

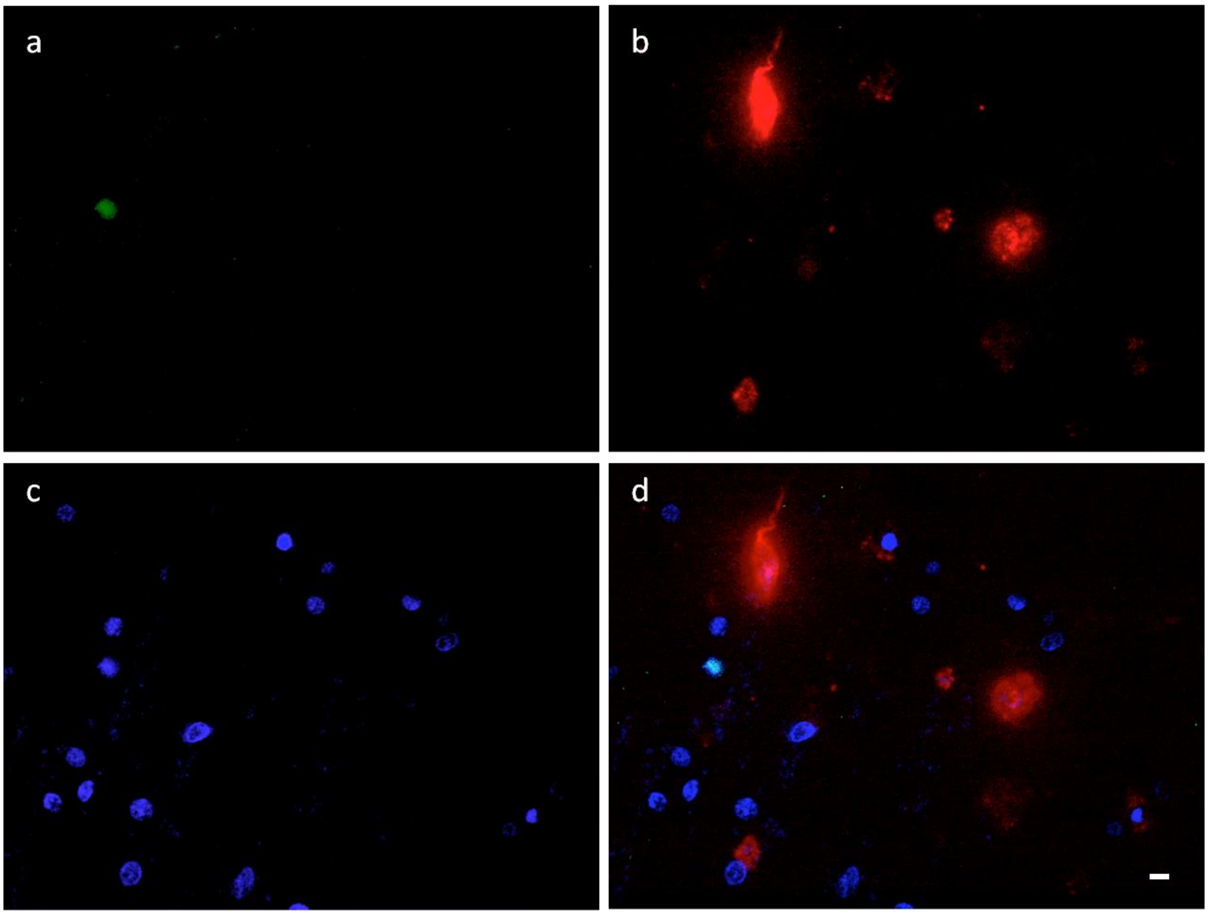

Fig. S1 Dual immunofluorescence with DAPI (nuclear) counter-stain, HSV-1 and TOMM70 (mitochondrial surface) antigen expression in HSE patient's brain tissue (cingulate gyrus): (a) green (HSV-1) channel; (b) red (TOMM70) channel; (c) blue (DAPI) channel; (d) composite image. Infected cell is labelled HSV-1 (green) but not by TOMM70 (red). HSV stain overlaps DAPI nuclear stain in infected cell. Cells in the area are TOMM70 positive (surrounding /overlapping DAPI stain). Scale bar 10μm.

Figure S2 Double immunoperoxidse staining for HSV-1 and CO1 in HSE patient.

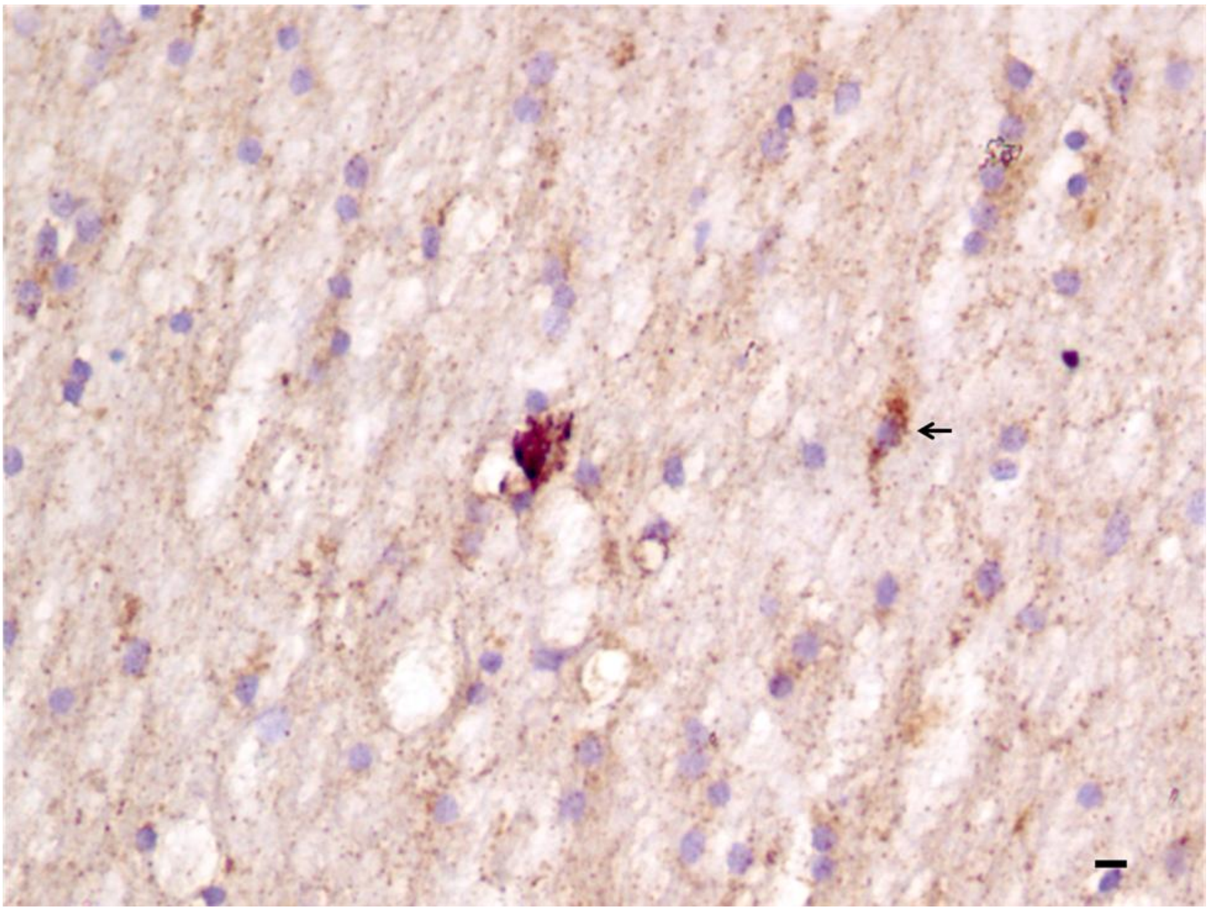

Fig. S2 Double immunoperoxidse staining for CO1 and then HSV-1 antigen expression in HSE patient's brain (cingulate gyrus). Occasional cells in the area (arrow) exhibit dense MT-CO1 labelled cytoplasm (brown [DAB]). High numbers of cells exhibit HSV-1 labelled nuclei (purple [VIP]) without dense CO1 labelled cytoplasm. Scale bar 10μm.

Material and Methods: Dual Immunofluorescence and double immunoperoxidase staining of brain tissue

Dual Immunofluorescence labelling for HSV-1 and Translocase of the outer mitochondrial membrane 70a (TOMM70) was performed on cingulate tissue sections from HSE subjects. A primary mouse anti-HSV-1 monoclonal antibody (clone 20.7.1; 1:100 dilution in TBST; Abcam) and primary rabbit anti-TOMM70a polyclonal antibody (Code: ab135602; 1:100 dilution in TBST; Abcam) were employed. Secondary anti-mouse polyclonal antibody conjugated with DyLight488 (1:200 dilution in TBST; AbDSerotec) and secondary anti-rabbit polyclonal antibody conjugated with DyLight549 (1:200 dilution in TBST; MenaPath) were used to obtain green and red fluorescence respectively. Nuclei were counterstained with blue fluorescent DAPI nucleic acid stain (Life-Technologies).

Double Immunoperoxidase staining of brain tissue

Sections of brain had previously been submitted for single immunohistochemistry for CO1 using a primary mouse polyclonal antibody (clone 1D6E1A8; 1:200 dilution in Tris buffered saline with 0.05% Tween 20 [TBST]; Abcam) and the reaction visualized using DAB chromogen, yielding a brown stain (Methods). Following single immunoperoxidase staining, the same sections were further stained with a primary rabbit anti-HSV1 polyclonal antibody (clone 20.7.1; 1:800 dilution in TBST; Abcam) followed by Envision anti Rabbit peroxidase conjugated revelation system (Dako). Second peroxidase reaction was developed using VIP (Vector Laboratories) yielding a purple stain. Nuclei were counterstained with haematoxylin.

Samples were analysed using an Eclipse 80i epi-fluorescent microscope (Nikon).
