# Supplementary material for: Herpes simplex encephalitis is linked with selective mitochondrial damage; a post-mortem and in vitro study
Source: Acta Neuropathol. 2016 Jul 25;132:433–51. doi: 10.1007/s00401-016-1597-2 (PMC4992034; doi:10.1007/s00401-016-1597-2)
Supplement: Supplementary file 3 — Supplementary material 3 (PDF 281 kb) [file 401_2016_1597_MOESM3_ESM.pdf]

File: ESM_3 (Online Resource 3)

Article Title: Herpes Simplex encephalitis is linked with selective mitochondrial damage; a post-mortem and in-vitro study.

Journal Name: Acta Neuropathologica

Authors: Małgorzata Wnęk, Lorenzo Ressel, Emanuele Ricci, Carmen Martinez, Julio Cesar Villalvazo Guerrero, Zarini Ismail, Patrick F. Chinnery, Colin Smith, Anja Kipar, Beate Sodeik, Tom Solomon, Michael J. Griffiths

Corresponding Author: Dr Michael J. Griffiths; e-mail: griffmj@liverpool.ac.uk; tel: +44-151-795-9657; fax: +44-151-795-5529

Fig.S3: GFAP and HSV-1 and antigen expression during *in vitro* HSV-1 infection.

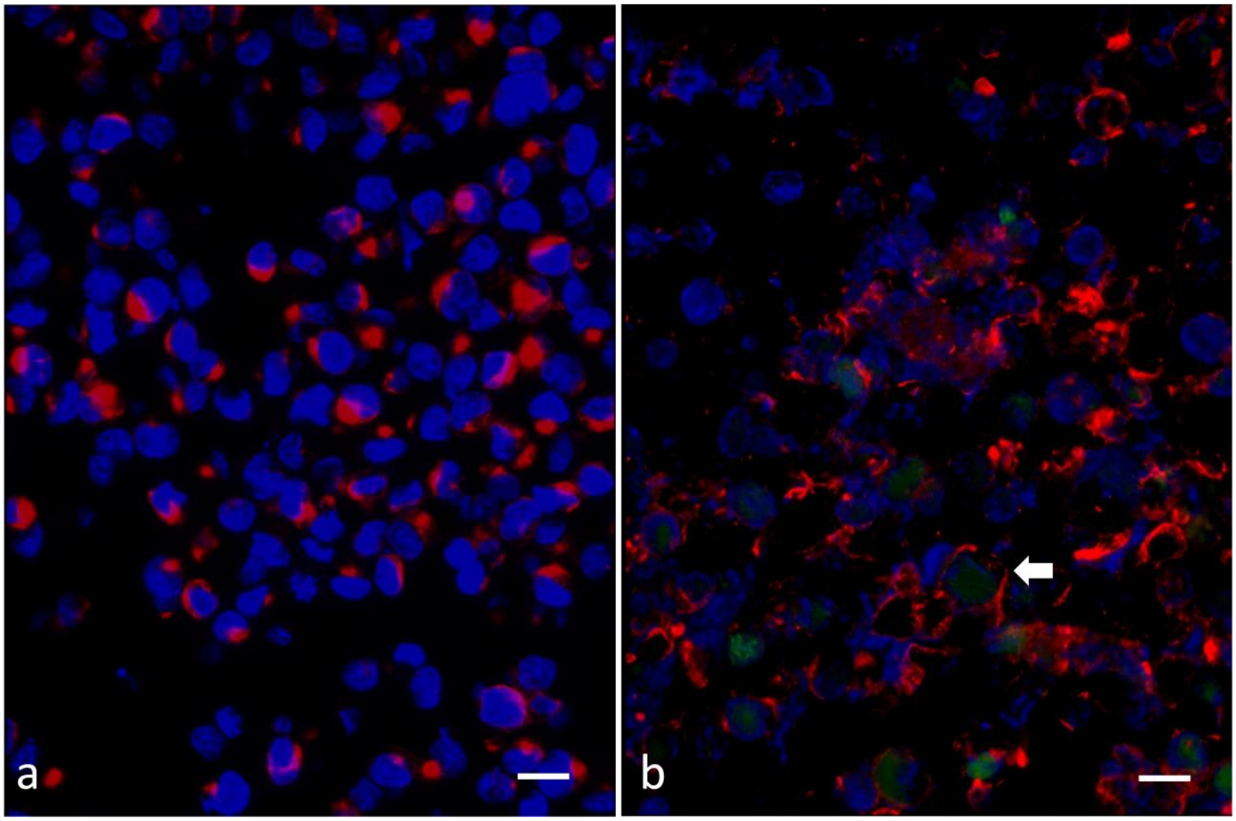

Fig.S3 Dual immunofluorescence for HSV-1 and Glial Fibrillary Acid Protein (GFAP) in astrocyte cultures: Non-infected and infected human astrocytes (recovered from the *in vitro* cultures at 48h pi) were labeled for GFAP and HSV-1 antigen, with DAPI nuclear counter-stain (blue): (a) Non-infected astrocytes; positive GFAP (red) expression is observed in the cytoplasm of all cells. (b), HSV-1 infected astrocytes: All cells express GFAP (red). HSV1 antigen expression (green) is observed in some cells (arrow). Scale bars 20µm.

Fig.S4: Inclusion bodies within cells recovered from astrocyte cultures

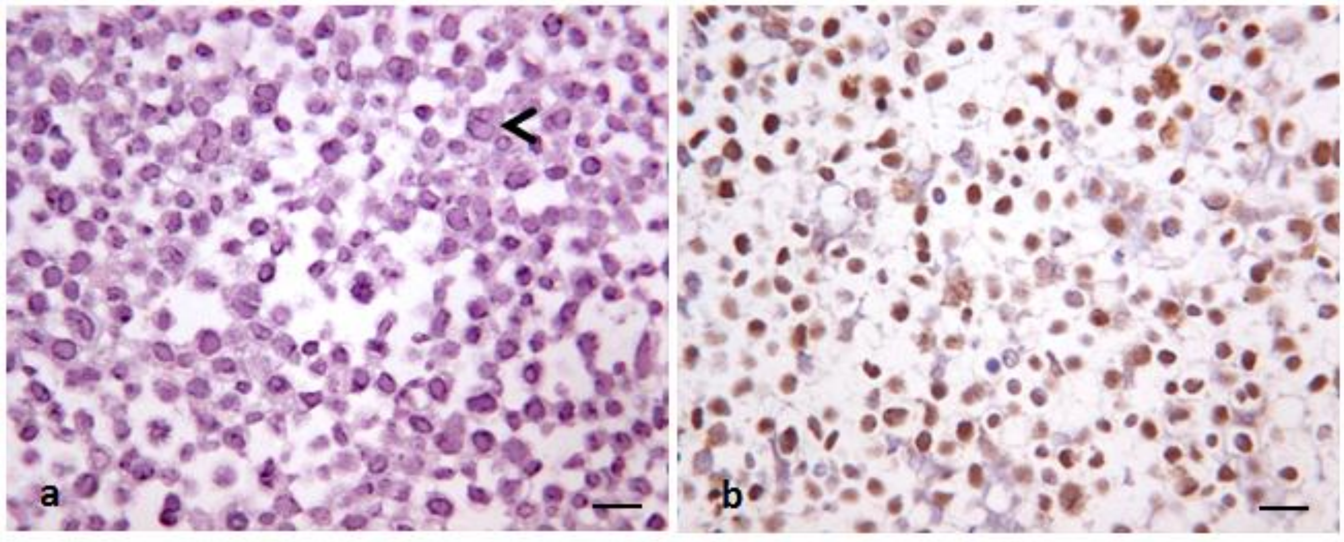

Fig.S4 Astrocytes recovered from *in vitro* HSV infection culture (48 hrs pi): (a) Hematoxylin & eosin stain – a high proportion of cells exhibit large pink/purple masses within the cell nuclei (viral inclusion bodies - arrow head); (b) Immunoperoxidase stain for HSV-1 antigen (visualized using DAB [brown]). Almost all cells exhibit dense positive staining for HSV-1 within the nucleus. Scale bar 20 µM.

Materials and Methods

GFAP / HSV1 dual immunofluorescence on astrocyte culture

A primary mouse anti-HSV-1 monoclonal antibody (clone 20.7.1; 1:100 dilution in TBST; Abcam) and primary rabbit anti-GFAP polyclonal antibody (1:100 dilution in TBST; Dako) were employed on astrocyte culture. Secondary anti-mouse polyclonal antibody conjugated with DyLight488 (1:200 dilution in TBST; AbDSerotec) and secondary anti-rabbit polyclonal antibody conjugated with DyLight549 (1:200 dilution in TBST; MenaPath) were used to obtain green and red fluorescence respectively. Nuclei were counterstained with blue fluorescent DAPI nucleic acid stain (Life-Technologies) and samples were analysed using an Eclipse 80i epifluorescent microscope (Nikon).
