# Supplementary material for: Herpes simplex encephalitis is linked with selective mitochondrial damage; a post-mortem and in vitro study
Source: Acta Neuropathol. 2016 Jul 25;132:433–51. doi: 10.1007/s00401-016-1597-2 (PMC4992034; doi:10.1007/s00401-016-1597-2)
Supplement: Supplementary file 4 — Supplementary material 4 (PDF 384 kb) [file 401_2016_1597_MOESM4_ESM.pdf]

File: ESM_4 (Online Resource 4)

Article Title: Herpes Simplex encephalitis is linked with selective mitochondrial damage; a post-mortem and in-vitro study.

Journal Name: Acta Neuropathologica

Authors: Małgorzata Wnęk, Lorenzo Ressel, Emanuele Ricci, Carmen Martinez, Julio Cesar Villalvazo Guerrero, Zarini Ismail, Patrick F. Chinnery, Colin Smith, Anja Kipar, Beate Sodeik, Tom Solomon, Michael J. Griffiths

Corresponding Author: Dr Michael J. Griffiths; e-mail: griffmj@liverpool.ac.uk; tel: +44-151-795-9657; fax: +44-151-795-5529

Figure S5 Relative transcript abundance for apoptotic mediators during in vitro HSV infection

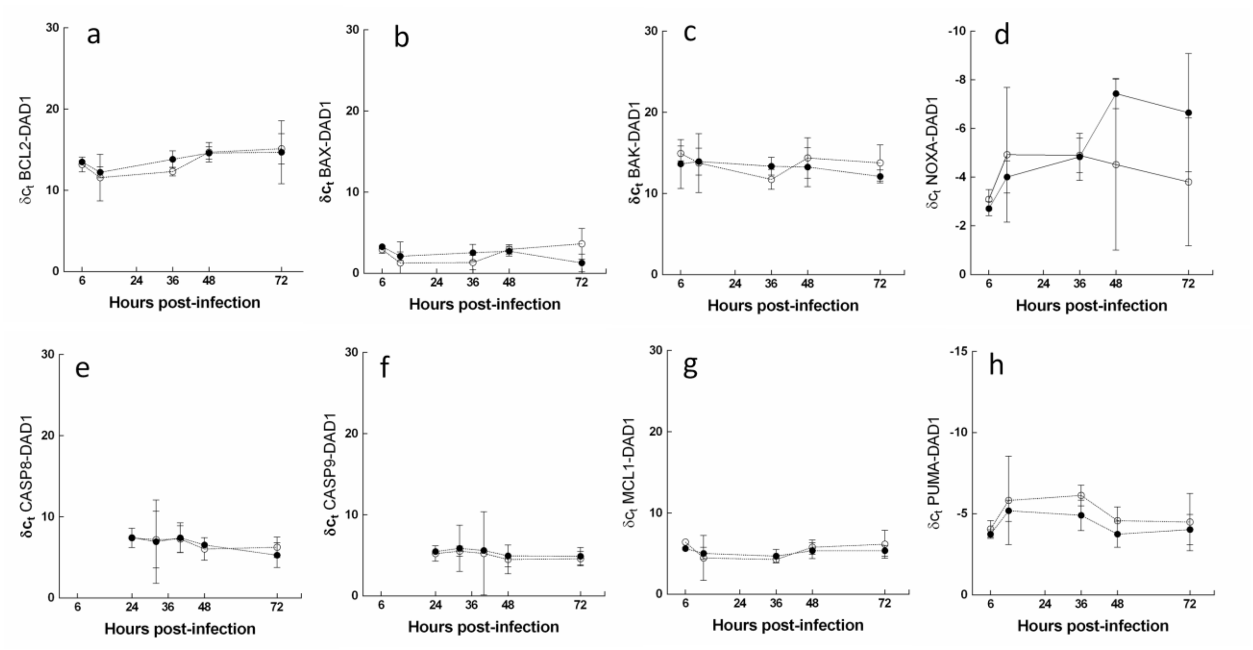

Fig. S5 Relative transcript abundance for apoptotic mediators (a) BCL2, (b) BAX, (c) BAK, (d) NOXA, (e) CASP8, (f) CASP9. (g) MCL1, (h) PUMA in HSV infected (black circles) compared to non-infected (open circles) astrocyte cultures. Y-axis - relative abundance expressed as δCt (difference in Ct compared to DAD1[relative invariant gene]). X-axis - time post infection in hours. Minimum of three replicates per target. Error bars - 95%CI. No significant changes in target transcript abundances were observed among infected compared to non-infected cells during the time-course.

Table S6: Viable cell counts in non-infected and HSV infected astrocyte cultures.

	**6h**	**24h**	**48h**	**72h**
**HSV-1 infected** Cell no. x 10^6/ml	1.0 (1-1)	0.79 (0.68-0.91)	0.63 (0.37-0.96)	0.57 (0.23-0.75)
**Non Infected** Cell no. x 10^6/ml	1.0 (1-1)	0.98 (0.85-1.1)	0.99 (0.94-1.07)	1.05 (0.89-1.15)

Mean and 95%CI (in brackets) of cell counts recovered from a minimum of three replicate astrocyte *in vitro* cultures during the HSV infection time-course at 6, 24, 48, 72h pi. Viable cells identified by remaining unstained following incubation with trypan blue (Method - below).

Materials and Methods

Viability staining and cell counting

Number of viable cells was evaluated using Trypan Blue staining. Cells were recovered from each well via scraping. Cells were pelleted via centrifuge at 1300rpm for 10mins. Supernatant (media) was removed and 500µl of sterile phosphate buffered saline (PBS) was added. Cells were briefly vortexed to re-suspend. An aliquot (10µl) from each suspension was further diluted 1in 10 (with 90µl PBS). Trypan blue (5µl; 0.4% [w/v]) was added to aliquots (45µl) from the original and 1/10 diluted cell suspensions. Suspensions were incubated at 37°C for 10 mins. Using 10µl aliquots, total (stained + unstained) non-viable (stained) and viable (unstained) cell counts were measured using a Haemocytometer. At least 3 wells per each exposure and time-point were counted. Mean and 95% CI (of the dilutions and replicates) were calculated.

Figure S6 Astrocyte morphology during *in vitro* culture.

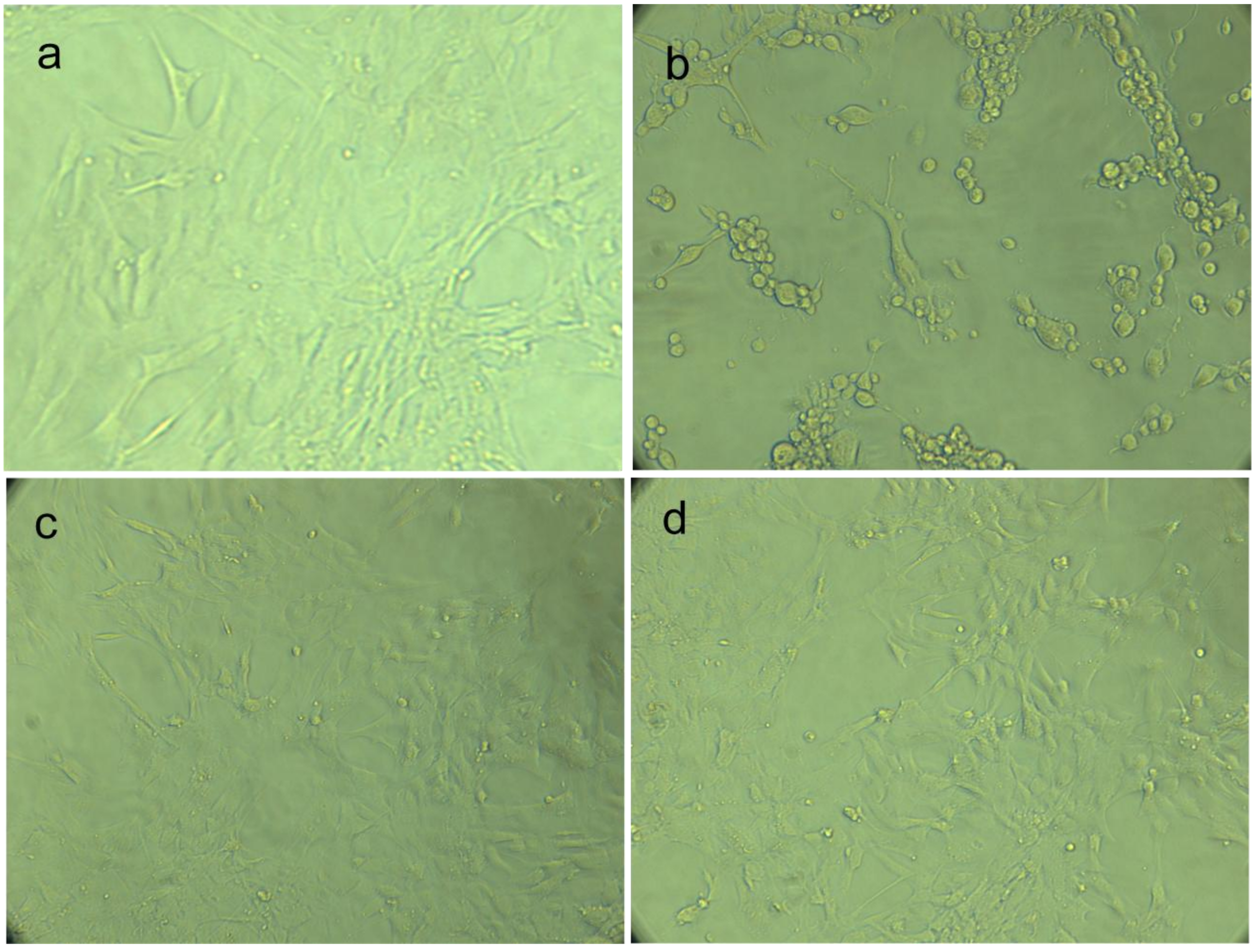

Fig. S6 In-vitro astrocyte cultures viewed in-situ using light microscopy (72h pi): (a) Non-infected astrocytes exhibiting typical stellate morphology; (b) HSV infected astrocytes (MOI 0.1) exhibiting globoid appearance (c) Non-infected Non-drug exposed (d) Non-infected minocycline exposed (60µM). Last two images show astrocytes exhibiting the same typical stellate morphology. Images obtained using CETI Inverso-TC 100 inverted microscope (20X magnification).
