# Supplementary material for: Herpes simplex encephalitis is linked with selective mitochondrial damage; a post-mortem and in vitro study
Source: Acta Neuropathol. 2016 Jul 25;132:433–51. doi: 10.1007/s00401-016-1597-2 (PMC4992034; doi:10.1007/s00401-016-1597-2)
Supplement: Supplementary file 5 — Supplementary material 5 (PDF 240 kb) [file 401_2016_1597_MOESM5_ESM.pdf]

File: ESM_5 (Online Resource 5)

Article Title: Herpes Simplex encephalitis is linked with selective mitochondrial damage; a post-mortem and in-vitro study.

Journal Name: Acta Neuropathologica

Authors: Małgorzata Wnęk, Lorenzo Ressel, Emanuele Ricci, Carmen Martinez, Julio Cesar Villalvazo Guerrero, Zarini Ismail, Patrick F. Chinnery, Colin Smith, Anja Kipar, Beate Sodeik, Tom Solomon, Michael J. Griffiths

Corresponding Author: Dr Michael J. Griffiths; e-mail: griffmj@liverpool.ac.uk; tel: +44-151-795-9657; fax: +44-151-795-5529

Table S7: Proportion of DAB or SDH staining cells in minocycline treated and non-treated HSV-1 infected astrocyte cultures.

	**24h**	**48h**
**Minocycline**<br>DAB proportion (CO activity)	0.62 (0.47-0.77)	0.73** (0.64-0.82)
**Minocycline**<br>DAB or SDH proportion (viable)	0.71 (0.57-0.85)	0.78* (0.65-0.85)
**Non treated**<br>DAB proportion (CO activity)	0.57 (0.49-0.66)	0.17 (0.07-0.27)
**Non treated**<br>DAB or SDH proportion (viable)	0.64 (0.53-0.75)	0.52 (0.40-0.64)

Table S7: Proportions of the total area of the astrocyte monolayer stained with DAB only (representing cells with CO function) and DAB or NBT stained (representing CO or SDH activity among adherent viable cells in monolayer) during the HSV-1 infection time-course at 24 and 48h pi were examined. Three researches (two blinded to exposure and time pi) analysed the images. Area was measured using ImageJ (see methods - main manuscript). Higher proportions of both DAB and 'DAB or NBT' stained cells were observed in the treated cultures.**Significantly larger proportion of monolayer surface stained positively for DAB at 48h pi among treated compared to untreated cultures ($p=0.029$). *Significantly larger proportion of monolayer surface stained positively for DAB or NBT at 48h pi among treated compared to untreated cultures ($p=0.046$). Mean and 95%CI (in brackets) presented. Significance assessed by Mann Whitney U test.

Figure S7: HSV1 replication following exposure to minocycline and/or aciclovir

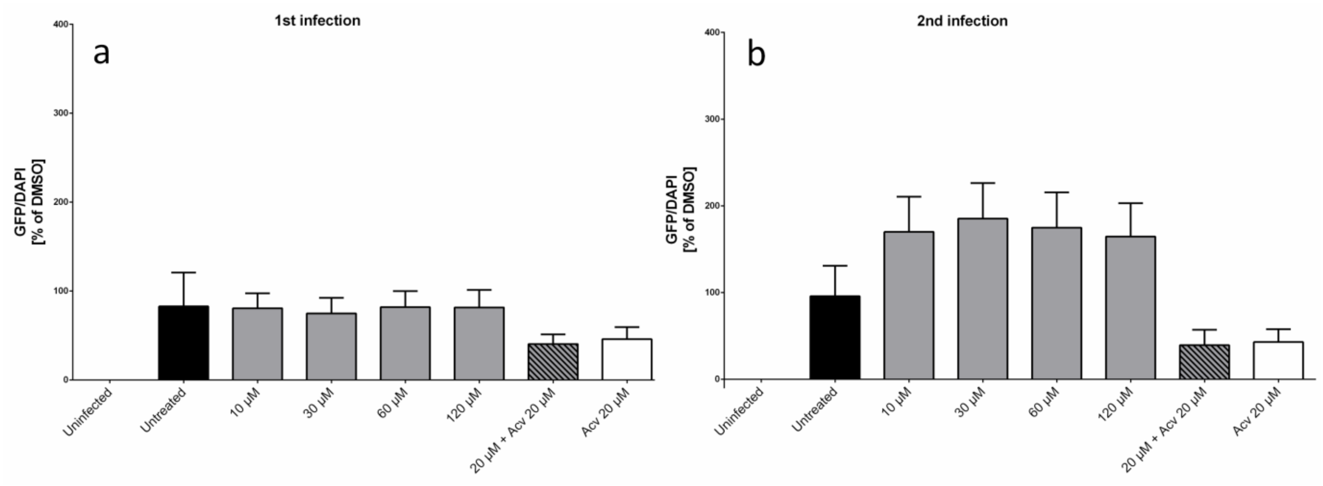

Fig. S7 Assessment of HSV1 replication following minocycline exposure via 2 phase infection using GFP labeled HSV. HeLaCNX cells were uninfected, infected but untreated, treated with minocycline alone (10, 30, 60 and 120µM), minocycline plus aciclovir (20µM for both) or aciclovir alone (20µM). Cells were infected with HSV1(17^+)Lox-$_{pMCMV}$GFP (3 x 10^5 pfu/ml). After 24h pi the supernatants from each well were used to infect fresh wells for 48 hrs. Average GFP fluorescence intensity per cell (GFP/DAPI) was determined at (a) 24h post 1st infection and (b) 48h post 2nd infection (of fresh cells). There was no significant difference in HSV fluorescence intensity at 24h pi between untreated and 60uM minocycline.

At 48h pi in the 2nd infection there was a significantly higher GFP-HSV fluorescence in the wells exposed to supernatant from the minocycline exposed cells compared to untreated ($p<0.001$). In the 2nd infection there was no significant difference in the wells previously exposed to minocycline plus aciclovir compared to aciclovir alone. Significant differences in fluorescence between exposures were assessed using the Mann Whitney U test.

Materials & Methods: Assessment of HSV-1 replication

To quantify HSV-1 replication, we used HSV1(17^+)Lox-$_{pMCMV}$GFP (HSV1-GFP for short), which expresses a soluble GFP transgene under the control of the murine cytomegalovirus (MCMV) major immediate-early promoter (Snijder et al. 2012); http://www.ncbi.nlm.nih.gov/pubmed/22531119). 3000 HeLaCNX cells per well of a 384-well plate were infected in quadruplicates at 3 x 10^5 pfu/ml of HSV1-GFP in the absence or presence of the indicated drugs. After 24h, the supernatants from each well were used to infect fresh wells for 48h. The cells of the first plates were fixed at 24h pi (hours post infection) and the ones from the second plate at 48h pi with 3% paraformaldehyde (PFA) in PBS, permeabilized with 0.1% Triton-X-100 and cell nuclei DNA were stained with 0.05 µg/ml 4',6-diamidino-2-phenylindole (DAPI) in PBS with 0.05% (vol/vol) DMSO, 0.0005% (vol/vol) NP-40, 0.025% (wt/vol) BSA, 0.05 mM Tris-HCl, pH 7.4, 0.73 mM NaCl, 0.01 mM CaCl2, and 0.11 mM $MgCl_2$ (Ivanova et al. 2016, http://www.ncbi.nlm.nih.gov/pubmed/27009950). We imaged HSV1-GFP-positive cells and the nuclei from 6 independent sites per well using a wide-field high-content fluorescence microscope fitted with a 10x objective (ImageXpress Micro; Molecular Devices, Biberach an der Riss, Germany). Images were automatically recorded, and the number of nuclei and the GFP fluorescence intensity per cell were determined using the image analysis software CellProfiler (Carpenter et al. 2006, http://www.ncbi.nlm.nih.gov/pubmed/17076895; Devadas et al. 2014, http://www.ncbi.nlm.nih.gov/pubmed/25210183).

Figure S8 Astrocyte viability following in vitro exposure to minocycline.

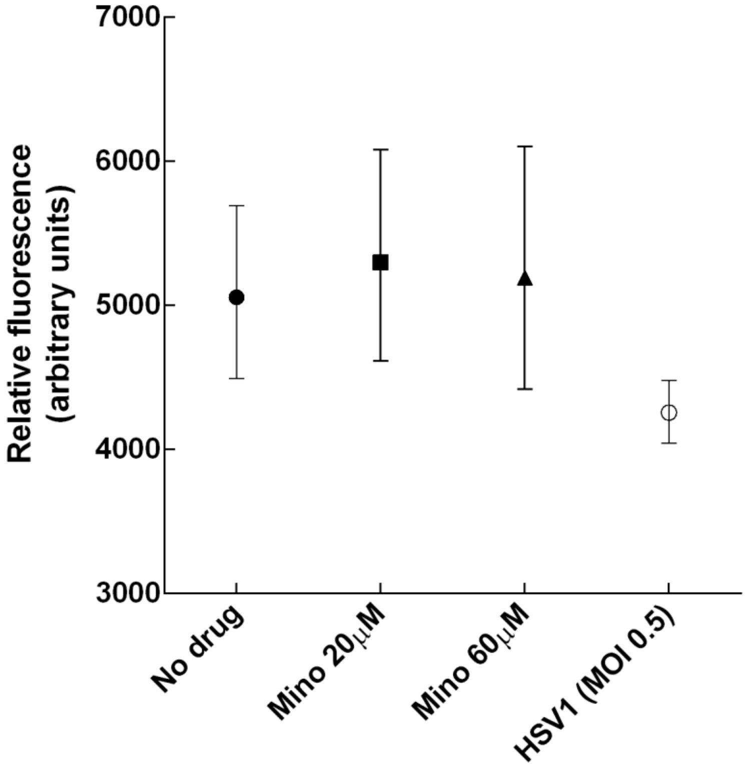

Fig. S8. Cell viability was indirectly assessed using Calcein AM fluorescence among astrocyte cultures +/- exposure to minocycline. Astrocyte monolayers ($2.5x10^5$ cells / well) were cultured in 96 well microplates (CoStar) over 72 hrs (Methods). Minocycline (20 or 60µM final concentration) was added each day. One set of cultures were not exposed to drug and one set was infected with HSV-1 at MOI 0.5 (positive control). After 72 hours the cells were incubated with Calcein AM (0.5µM final concentration) for 30 minutes, following the manufacturer's protocol (R&D Systems). Average fluorescence (background subtracted) within each well was measured via a plate reader (Fluostar Omega [BMG Labtech]) using a 485 nm excitation / 520 nm emission filter. The geometric mean fluorescence was plotted for each set of cultures (minimum of 4 replicate cultures per set). Error bars indicate 95% CI. Drug and non-drug exposed cultures showed no significant difference in cell fluorescence. HSV-1 infected cells exhibited significantly lower fluorescence, indicating reduced cell viability and/or cell number, compared to non-drug exposed (p=0.02). Significant differences in fluorescence between exposures at 72 hours were assessed using the Mann Whitney U test.

Figure S9 Astrocyte cell number following in vitro exposure to minocycline.

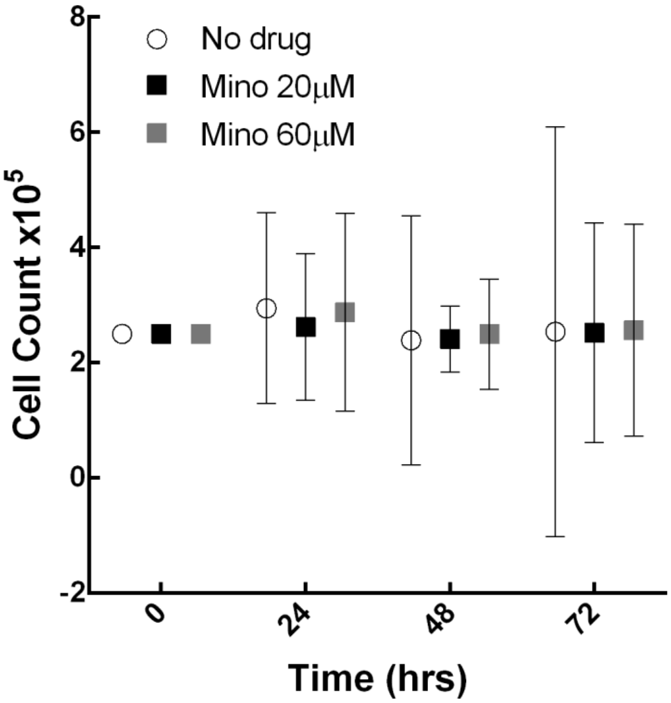

Fig. S9 Astrocyte cell number following in vitro exposure to minocycline. Astrocyte number was directly assessed by manual counting. Astrocytes ($2.5x10^5$ cells / well) were cultured in 96 well microplates (CoStar) over 72 hrs (Methods). Minocycline (20 or 60μM final concentration) was added each day. Each day pairs of cultures were recovered by scraping. Cells from the resulting suspension were incubated with trypan blue (methods; Online resource 4). Viable cells counted using a haemocytomter. Mean and 95%CI intervals presented. No change in viable cell number was observed in drug exposed compared to non-drug exposed cultures. There was also no significant difference in total cell numbers (not shown).
